# Bringing Molecules Together: Synergistic Coadsorption at Dopant Sites of Single Atom Alloys

**DOI:** 10.1021/jacs.4c07621

**Published:** 2024-10-02

**Authors:** Fabian Berger, Julia Schumann, Romain Réocreux, Michail Stamatakis, Angelos Michaelides

**Affiliations:** † Yusuf Hamied Department of Chemistry, University of Cambridge, CB2 1EW Cambridge, U.K.; ‡ Thomas Young Centre and Department of Chemical Engineering, University College London, WC1E 7JE London, U.K.; ¶ Department of Chemistry, University of Oxford, OX1 3QZ Oxford, U.K.

## Abstract

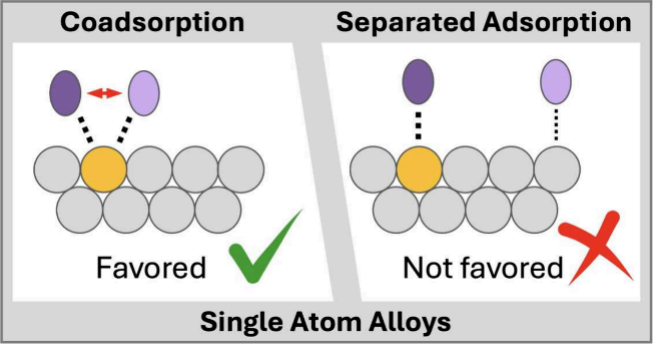

Bringing molecules together on a catalytic surface is a prerequisite for bimolecular and recombination reactions. However, in the absence of attractive interactions between reactants, such as hydrogen bonds, this poses a challenge. In contrast, based on density functional theory, we show that coadsorption at active sites of single-atom alloys (SAAs) is favored and that coadsorption is a general phenomenon observed for catalytically relevant adsorbates on a broad range of SAAs under temperature and pressure conditions commonly employed for catalysis. Dopants located in both terrace sites and in step edge defects exhibit a preference for coadsorption, displaying similar periodic trends. Using kinetic Monte Carlo simulations, we compare the reactivity of a model reaction on both a pure metal and an SAA and show that the preference for coadsorption significantly alters the overall reaction energy profile, even when the barriers for the rate-determining elementary step are identical. In our models, the coadsorption preference enhances the catalytic activity of the SAA surface by several orders of magnitude compared to the pure metal. We also report infrared (IR) spectroscopic signatures of coadsorption, which facilitate experimental detection. Analysis reveals that in these systems repulsive lateral interactions between nearby molecules are more than compensated for by the enhanced binding at dopant sites. Among the broad range of systems considered, SAAs containing early transition metals (TMs) exhibit the strongest coadsorption preference, which can be rationalized by assuming the existence of an optimal number of electrons involved in binding. The strong coadsorption preference, together with facile product desorption from early TMs, renders these systems attractive candidates for catalysis. Moreover, these SAAs could open new routes for reduction reactions because coadsorption with hydrogen is favored.

## Introduction

The development of improved catalysts for industrially relevant processes has become a pivotal task for chemists, as most currently used catalysts were developed in the 20th century, a time when climate change was not a prominent concern. Designing new catalysts requires a reliable and accurate understanding of fundamental reaction mechanisms and important details including the barriers of rate-determining steps, adsorption of reactants, and desorption of products.

A major challenge in catalysis is the high stability and thus inertness of common chemical feedstocks. Activating unreactive molecules requires highly active catalysts, which typically comes at the cost of reduced selectivity. The novel material class of single-atom alloy (SAA) catalysts^1−6^ offers a promising solution to this activity-selectivity trade-off, although it is still far from being fully understood. SAAs are generally composed of catalytically highly active elements atomically dispersed in less active yet more selective host metals. Alloying metals in this way leads to unique properties that can break the linear scaling relationship between selectivity and activity obeyed by traditional metal catalysts.^7,8^ Lower reaction barriers and thus increased activity typically correlate with strong adsorption of products, hampering their release via desorption and compromising the traditional catalyst’s selectivity. SAAs can overcome this hurdle by allowing reaction intermediates or products to spill over from active dopant sites onto the surface of the host metal, introducing bifunctionality.^1^ This enables significantly enhanced catalytic activity compared to traditional metal surfaces without impeding product selectivity.^9,10^

While the stabilization of transition states and the associated lowering of reaction barriers is a key property of catalysts, another essential aspect is their ability to bring reactants together at an active site in a reaction-ready *coadsorbed state*. Naturally, molecules such as CO tend to remain apart from each other due to repulsive lateral interactions when no hydrogen bonding or strong dispersion interactions are involved. Increasing the reactant pressure could address this issue, but it would come at an increased energetic cost and additional practical challenges. Therefore, approaching this problem from a materials perspective can help develop more sustainable processes.

Nanoporous materials such as metal–organic frameworks (MOFs) and zeolites^11−14^ as well as the active pockets of enzymes^15^ utilize steric constraints for catalysis. On metal surfaces and nanoparticles, however, there are no steric constraints to impose such a coadsorbed state. Introducing active sites capable of interacting strongly with nearby adsorbates can enforce the proximity of adsorbates by overcompensating their repulsive lateral interactions. This approach has been utilized, for example, in the coadsorption of CO molecules (dicarbonyl species) on a single-atom catalyst (SAC) consisting of Rh^+^ ions dispersed on γ-Al_2_O_3_.^16^

Coadsorption at formally neutral metallic dopant sites in alloys would not necessarily be expected, as adsorbates such as CO are known to exhibit strong repulsive lateral interactions.^17−21^ In this vein, such species have not been reported for CO adsorption on RhCu(111)^22^ and NiCu(111).^23^ However, this cannot be the whole picture, as the surprising observation of CO coadsorbed at the same dopant site on CoCu(110)^24^ and RhCu(100)^25^ shows. Recent computational work also suggests that up to four CO molecules can adsorb on certain Cu-based 3d transition metal (TM) SAAs.^26^ This disparity illustrates the complexity of the coadsorption phenomenon. While there are many excellent experimental and simulation studies on SAAs,^27−33^ our apparent lack of understanding of coadsorption motivates a comprehensive investigation in the presented work. The aim is to unravel the fundamental principles dictating which dopants facilitate such coadsorption motifs and which adsorbates can be coadsorbed at a dopant.

**Figure 1:**
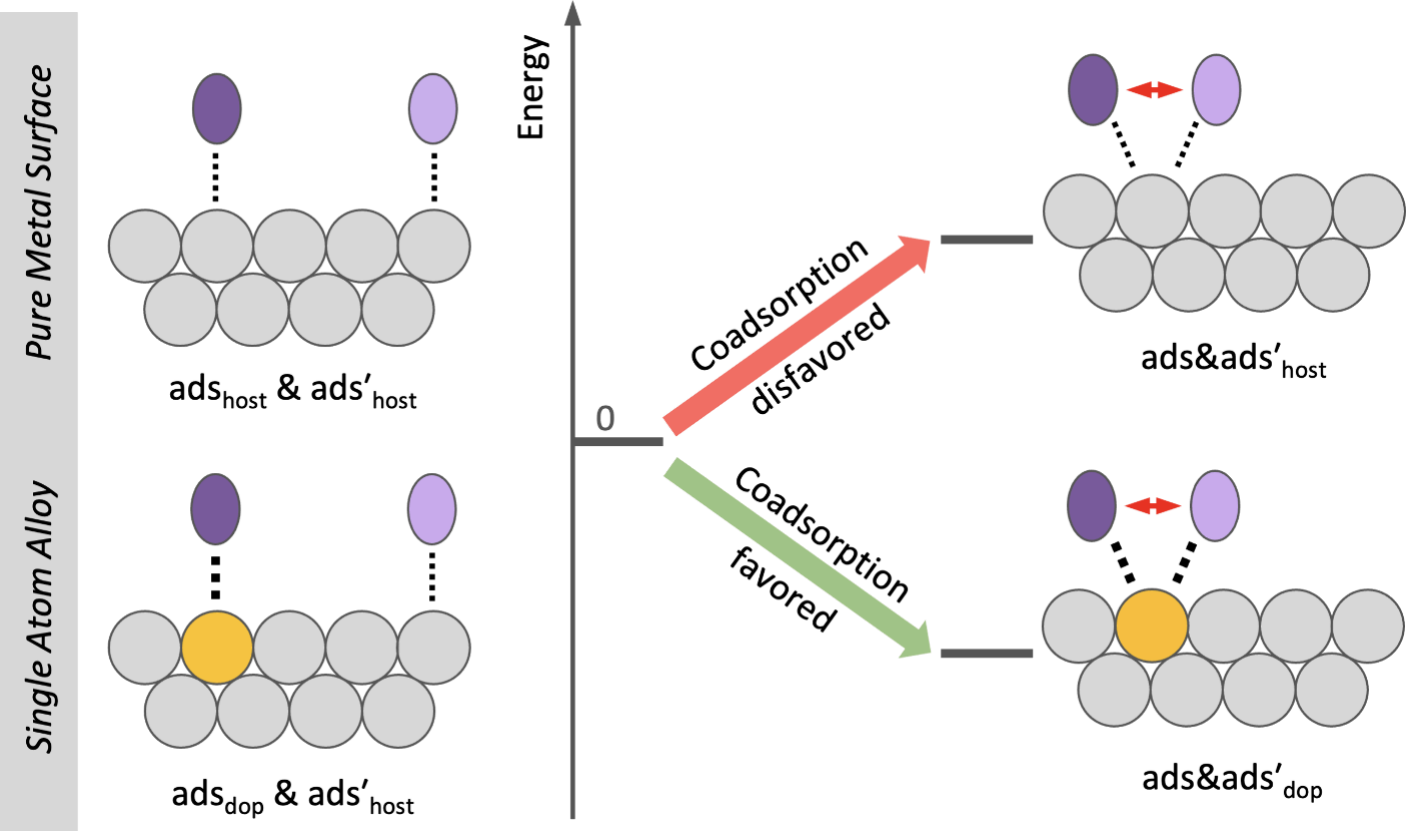
Schematic representation of the adsorption motifs for adsorption at separated sites (left) as well as for coadsorption at the same atom (right). Adsorbates (purple ovals) can adsorb on a pure metal (gray circles) surface or at a dopant site (yellow circle) of a single atom alloy (SAA). Potential repulsive lateral interactions between adsorbates (red arrows) and attractive adsorbate–surface interactions (dotted lines) are indicated. On a pure metal, coadsorption of, e.g., CO at the same atom is disfavored; at a dopant atom of an SAA, it is generally favored.

In this study, we demonstrate for a selection of catalytically relevant adsorbates and a broad range of SAAs that coadsorption at one site is generally favored. This preference, which is schematically illustrated in Figure 1, holds for dopants located in terrace sites and for those incorporated into step edge defects. The phenomenon arises from the highly localized energetic stabilization of adsorbates in the proximity of chemically reactive dopant atoms that are incorporated into inert, weakly interacting host metal surfaces. We first discuss the preference for coadsorption and highlight trends observed among SAAs based on 4d TM dopants in two scenarios: (1) High-pressure case: Adsorbates occupy all accessible dopant sites, requiring additional adsorbates to be located on the host metal surface. This is the most important scenario for real applications due to the low concentration of dopant atoms in SAAs and the high reactant pressures typically desired for catalysis. We find that CO coadsorption at the same dopant can be favored for all SAAs for which we have calculated phase diagrams, and that this is the predominant state even under relatively mild pressure and temperature conditions in all but the earliest and latest TMs. (2) Low-pressure case: All adsorbates are localized at dopant sites without needing to adsorb on the weakly interacting host metal surface. Although less relevant for practical applications, this scenario helps us to better understand the driving forces for coadsorption. To analyze the origin of this preference further, we employ a many-body decomposition of the adsorption energy, revealing that repulsive lateral interactions between coadsorbates are more than compensated for by the enhanced binding with the reactive dopant metal site. By employing kinetic Monte Carlo (KMC) simulations^34−37^ for a model reaction on a metal surface and an SAA, we demonstrate that the (de)stabilization of the coadsorption state at the active dopant site affects the overall reaction energy profile and thus the reaction kinetics by several orders of magnitude, even without assuming a change in the barrier of the rate-determining elementary reaction step. We also explore the applicability of the 10-electron count rule^38^ for molecules coadsorbed at a dopant and simulate the infrared (IR) spectroscopic signatures for the different adsorption motifs to aid experimentalists in identifying and assigning these species.

## Results and Discussion

The main objective of this work is to understand how easily molecules can be brought together at the active dopant sites of SAAs. We investigate this for combinations of the three fairly small adsorbates CO, NO, and H. These combinations consist of pure pairs (CO&CO, NO&NO, H&H), mixed pairs (CO&NO, CO&H, NO&H), and, due to its small size, H&H&H. We chose nine 4d transition metals (Y, Zr, Nb, Mo, Tc, Ru, Rh, Pd, Ag) as dopants to identify periodic trends, which are discussed in the main text. Additionally, two 3d (Ni, Cu) and two 5d (Ir, Pt) TMs are reported in the Supporting Information as important examples that provide insight into the effects of different periods. To understand the influence of the host metal, we consider ideal terrace sites of two commonly used coinage metal surfaces: Cu(111) and Ag(111), alongside step edge defects modeled by a Cu(211) surface. This defect type is not only one of the most common, but dopants have also been found to exhibit a certain preference for localization at step edges.^30^ The adsorbates are selected based on their specific properties and importance for catalysis. For instance, CO exhibits strong lateral interactions and can act as a catalyst poison.^39^ Furthermore, the adsorbates investigated (CO, NO, H) are relevant as reactants, intermediates, side- or byproducts in industrial processes^40−44^ or as part of (vehicle) exhaust emissions.

The key quantities that we discuss throughout the article are the adsorption energy, *E*_ads_, and the preference for coadsorption, *ΔE*_coads_. The contributions needed to calculate an adsorption energy
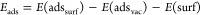
1are the energies of the adsorbate in the gas phase surrounded by vacuum, *E*(ads_vac_), the energy of the bare unloaded surface, *E*(surf), and the energy of the adsorbate adsorbed on the surface, *E*(ads_surf_). We consider two possibilities for adsorption on a surface: Adsorbates can be located either on a host or on a dopant site. To calculate the preference for coadsorption
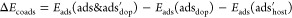
2for two identical or different adsorbates (ads and ads′), the adsorption energy of both adsorbates coadsorbed at the dopant site, *E*_ads_(ads&ads_dop_^′^); the adsorption energy of the adsorbate ads, individually adsorbed at a dopant site, *E*_ads_(ads_dop_); and the other adsorbate ads′, individually adsorbed at the host surface, *E*_ads_(ads_host_^′^), are required.

For each of the mixed adsorbates, the most stable combination of reference states is used, i.e., predominantly CO_host_&NO_dop_, H_host_&CO_dop_, and H_host_&NO_dop_. Details on the site preferences for the reference states, i.e., individual adsorption of the adsorbates on the pure metal surfaces and on SAAs, are compared to the literature^45−48^ in Section S2 of the Supporting Information. Adsorption energies and preferences for coadsorption of all investigated systems are provided in Sections S2 and S3, respectively, and settings used for the DFT calculations with the RPBE^49^ functional are described in Section S1 of the Supporting Information.

### Coadsorption at the Same Dopant Is Favored over Separated Adsorption

**Figure 2:**
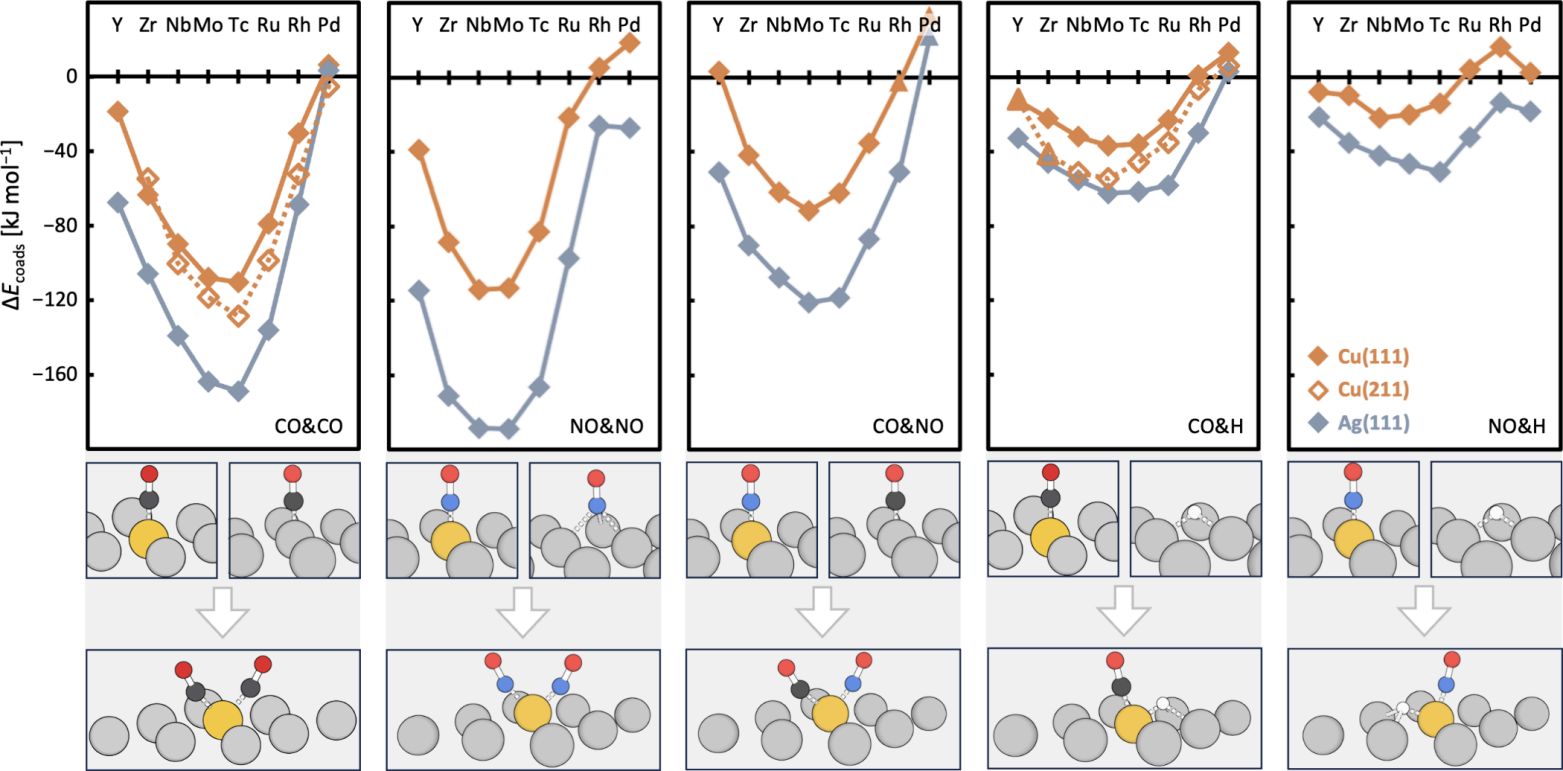
Preference for coadsorption when the adsorbate coverage exceeds the dopant concentration, *ΔE*_coads_, on SAA terrace sites modeled by Cu(111) (solid, orange lines) and Ag(111) (solid, gray lines) and at step edge defects modeled by Cu(211) (dotted, orange lines). *ΔE*_coads_ is defined as the difference of the adsorption energies between coadsorption at the dopant site and the most stable combination of individual adsorption at the dopant site accompanied by individual adsorption of the other adsorbate on the host metal surface; see eq 2. Negative values indicate a preference for coadsorption. Beneath each panel, a representative pair of individually adsorbed adsorbates and a coadsorption structure on the Ag(111) surface are shown. Symbols: CO_host_&CO_dop_, NO_host_&NO_dop_, CO_host_&NO_dop_, H_host_&CO_dop_, and H_host_&NO_dop_ – diamonds; CO_dop_&NO_host_, and H_dop_&CO_host_ – triangles. Color code: dopant element – yellow; Ag – gray; C – black; H – white; N – blue; O – red. Connecting lines are for illustrative purposes only.

Figure 2 shows the preferences for coadsorption, assuming all dopant sites are already occupied by one adsorbate. In general, the coadsorption tendencies on Cu and Ag SAAs reveal several key points:

(i) Preference for coadsorption: For almost all systems, coadsorption at the dopant is more stable than separated adsorption, where one adsorbate is at a dopant site and the other is weakly adsorbed on the host metal surface. The preference for coadsorption at the same dopant reaches up to −169 kJ mol^–1^ for CO&CO at Tc, −189 kJ mol^–1^ for NO&NO at Mo, −121 kJ mol^–1^ for CO&NO at Mo, −62 kJ mol^–1^ for CO&H at Mo, and −51 kJ mol^–1^ for NO&H at Tc on Ag(111)-based SAAs;
(ii) Periodic trends: There is a clear trend across the periodic table in coadsorption preference. While very early and late TMs exhibit the weakest preference for coadsorption (least negative), this adsorption motif remains favored (still negative) for all SAAs apart from some Ru, Rh, and Pd-based SAAs (positive). Coadsorption is most favored for early and central TMs, resulting in U-shaped curves that are more pronounced for coadsorption of CO and NO and less so for coadsorption with H; and
(iii) Comparison of surfaces: While the periodic trends are similar on Ag and Cu surfaces, the preference for coadsorption at the dopant is stronger on the Ag-based SAAs. This is due to the weaker adsorption on the more inert Ag host surface compared to the less inert Cu surface.^50^ Coadsorption at dopants located in step edges, as investigated for CO&CO and CO&H, is similar to that observed on terrace sites, with coadsorption at the defects being slightly more preferred. This increased preference is due to the more enhanced adsorption on undercoordinated, more reactive dopants located at step edges compared to adsorption at the corresponding undercoordinated, less reactive Cu atoms.

Focusing on CO coadsorption, a limiting case with strong repulsive lateral interactions, it can be stated that: *On pure metal surfaces, separated adsorption of CO is favored over coadsorption at the same atom, whereas on SAAs, CO coadsorption at one dopant is generally favored if the number of adsorbates exceeds the number of accessible dopant sites.*

### SAAs Containing Early TMs Can Stabilize Coadsorption at the Same Dopant Even in the Presence of Vacant Dopant Atoms

The preference for coadsorption in systems with no further vacant dopant sites is driven by the weak interactions of adsorbates with inert host metal surfaces, which push the adsorbates toward dopant sites. At these dopant sites, the stronger adsorbate-dopant interactions overcompensate for the potentially repulsive lateral interactions.

To understand the underlying driving forces for coadsorption better, we also investigate the scenario where vacant dopant sites are present. In this case, the preference for coadsorption, *ΔE*_coads_^vacant^, is defined as follows:
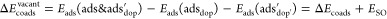
3In contrast to eq 2, the reference adsorption energies are not the energy of an adsorbate, ads, adsorbed at a dopant site accompanied by another adsorbate, ads′, adsorbed on the host surface. Instead, they are the adsorption energies of both adsorbates, ads and ads′, each individually adsorbed at separate dopant sites, *E*_ads_(ads_dop_) and *E*_ads_(ads_dop′_^′^), respectively. *ΔE*_coads_^vacant^ can also be expressed in terms of *ΔE*_coads_ and the spillover energy, *E*_SO_,^51^ of ads′ from the dopant site dop′ to the host surface. Hence, weak adsorption on the inert host surface cannot be the thermodynamic driving force for the coadsorption of adsorbates at dopant sites.

**Figure 3:**
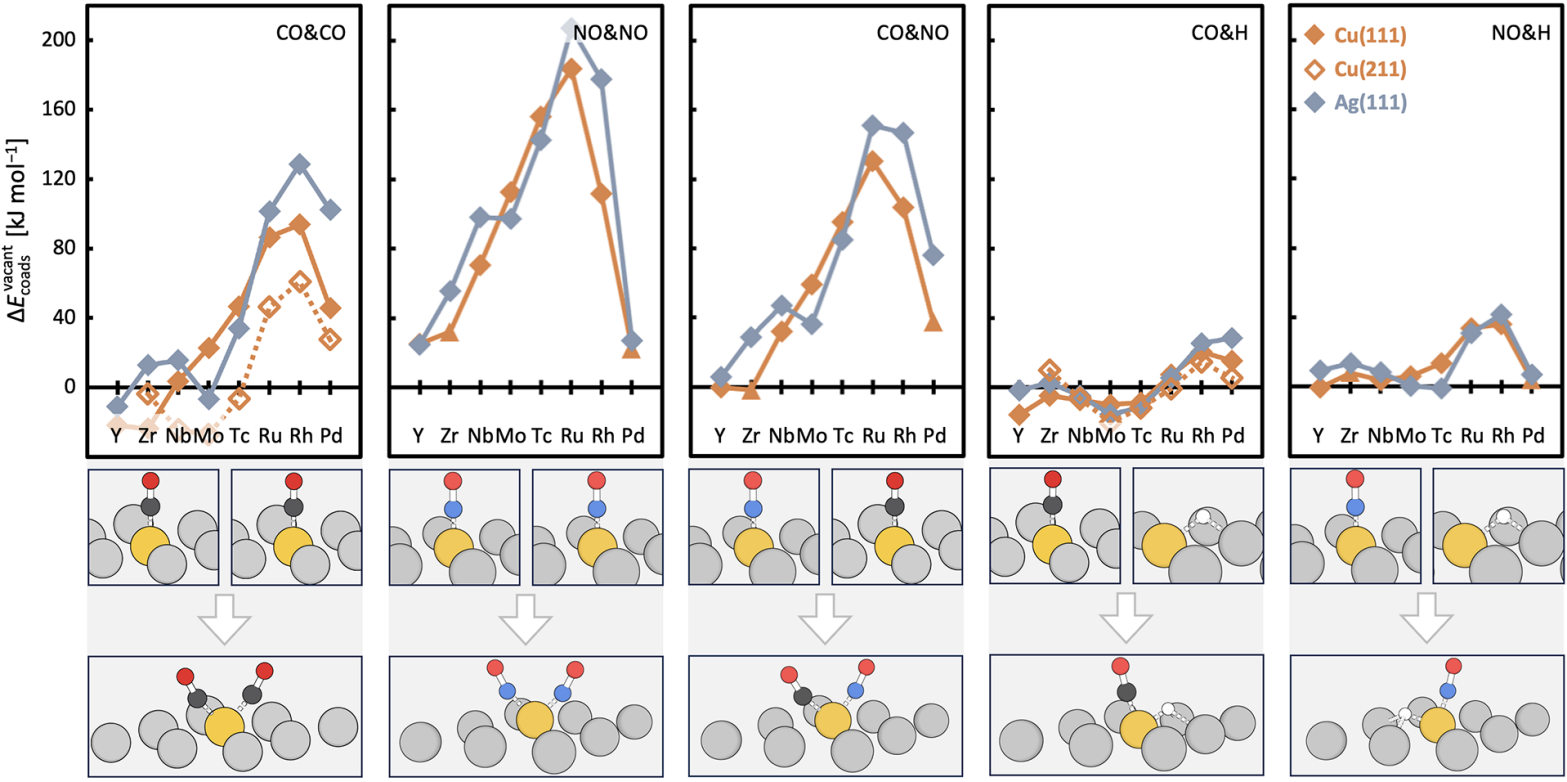
Preference for coadsorption when the adsorbate coverage is less than the dopant concentration, *E*_coads_^vacant^, on SAA terrace sites modeled by Cu(111) (solid, orange lines) and Ag(111) (solid, gray lines) and at step edge defects modeled by Cu(211) (dotted, orange lines). *E*_coads_^vacant^ is defined as the difference of the adsorption energies between coadsorption at the dopant site and both adsorbates individually adsorbed at a dopant site; see eq 3. Negative values indicate a preference for coadsorption. Beneath each panel, a representative pair of individually adsorbed adsorbates and a coadsorption structure on the Ag(111) surface are shown. Individual adsorption of H on a fcc hollow site as well as of CO and NO in atop position at the dopant–diamonds; individual adsorption of NO on a fcc hollow site of the dopant – triangles. Color code: dopant element – yellow; Ag – gray; C – black; H – white; N – blue; O – red. Connecting lines are for illustrative purposes only.

Figure 3 shows the coadsorption tendencies on the Cu and Ag SAAs, assuming that vacant dopant sites are present. Bringing molecules together at a dopant site is naturally more challenging under such circumstances. Surprisingly, however, coadsorption is indeed possible. While separated adsorption of CO and NO is generally favored over coadsorption at a dopant in a terrace site, this preference decreases for early TM dopants, resulting in coadsorption reaching similar stability to separated adsorption in some cases. The coadsorption of CO with H at the same dopant is predicted for some early and central TM dopants, such as Nb, Mo, and Tc. Although the coadsorption of NO with H is generally not favored, it is essentially thermoneutral for all SAAs, except for those with Ru, Rh, and Pd dopants, for which it is slightly endoenergetic. Coadsorption at dopants located in step edge defects follows the periodic trends observed at terrace sites closely for CO&H and more loosely for CO&CO, with the latter exhibiting a more pronounced preference for coadsorption.

The origin of the preferred formation of the coadsorption motif, even when vacant dopant sites are present, is explained in more detail in the section on the 10-electron count rule.^38^ According to this rule, which suggests that the optimal number of electrons involved in binding is 10, adsorbing a single adsorbate on an electron-poor early TM dopant does not necessarily bring the active site close to the most stabilizing electron count. Adding a second adsorbate to the same dopant site, i.e., forming the coadsorption motif, increases the number of electrons involved in binding. While this typically overshoots the optimal electron count for late TMs, rendering coadsorption less stable than individual adsorption, it can bring active sites consisting of early TMs closer to the optimal electron count. Consequently, coadsorption can become even more stable than individually adsorbing adsorbates at separate dopant sites.

Before concluding this section, we note that the trends observed, although very pronounced, are obtained using the RPBE functional. It is well-known that DFT adsorption energies can vary significantly depending on the exchange-correlation functional used.^52^ To ensure that these trends are not an artifact of a particular functional, we demonstrate in Section S4 of the Supporting Information that they persist across a selection of diverse^52^ DFT functionals, including RPBE,^49^ PBE,^53^ PBEsol,^54,55^ and optB86b-vdW.^56^ Furthermore, we assess the adequacy of the foundation machine learning potential MACE-MP-0,^57^ which is based on the MACE architecture^58^ and trained on PBE data from the Materials Project (MP).^59^ While this model performs well for the dopant elements Zr, Tc, Ru, Rh, and Pd, it exhibits poor performance for the dopant element Mo; see Section S4 in the Supporting Information for details.

### Many-Body Decomposition: Pairwise Adsorbate–Substrate Interactions Dominate Adsorption Energies

**Figure 4:**
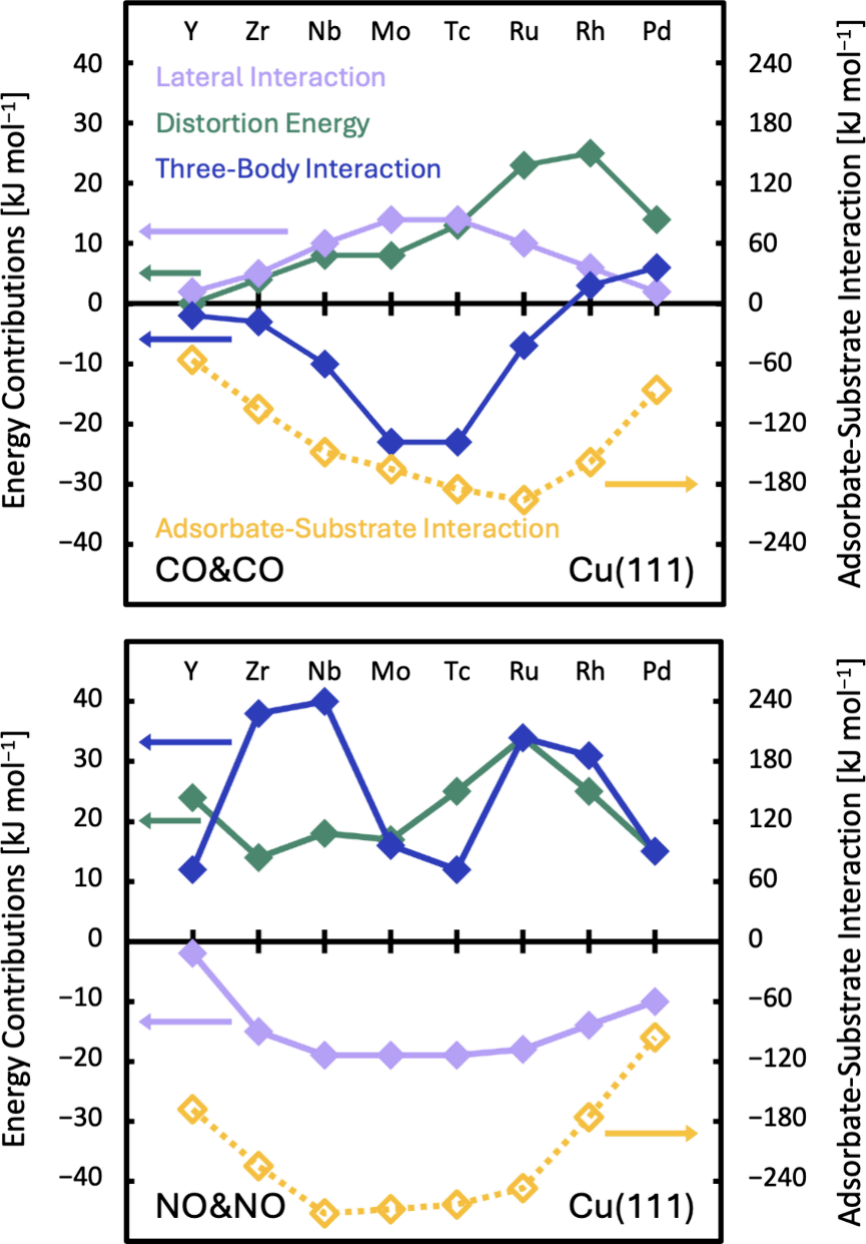
Many-body decomposition reveals that the adsorbate–substrate interactions dominate the adsorption energy. Contributions to the adsorption energies for the coadsorption of CO&CO (top) and NO&NO (bottom) on Cu(111) SAAs. Color code: green – summed one-body contributions (distortion energies); yellow – summed two-body contributions (adsorbate–substrate interactions); purple – two-body contribution (lateral adsorbate interaction); blue – three-body contribution. Connecting lines are for illustrative purposes only.

The origin of the preference for coadsorption is further investigated using a many-body decomposition of the adsorption energy. In this approach, the two adsorbates and the metal slab are each considered as one body. The analysis provides insights into the effect of all contributions: the distortion energies (one-body terms), the adsorbate–substrate interactions (two-body terms), the lateral interaction between the adsorbates (two-body term), and the higher-order contribution (three-body term). Figure 4 shows the contributions for CO&CO and NO&NO coadsorption. Details, including definitions and equations for the contributions, are provided in Section S5 of the Supporting Information.

Most of the adsorption energy originates from the adsorbate–substrate interactions (yellow diamonds in Figure 4), which explain 90% of the variation in adsorption energies; see Figure S4 in the Supporting Information. The lateral interactions (purple diamonds in Figure 4) are repulsive for the coadsorption of CO&CO but attractive for NO&NO. For both adsorbate types, the strength of lateral interactions increases as the distance between the adsorbates decreases. Since the distances between the adsorbates tend to be smallest for central TMs, as shown in Figure S3 in the Supporting Information, the lateral interactions tend to be strongest for central TMs. The qualitative differences between the lateral interactions can be explained by the unfavorably aligned dipoles for CO coadsorption and by the exothermic formation of a covalent bond via radical pairing of two coadsorbed NO molecules.^7,60,61^

The three-body contributions (blue diamonds in Figure 4) tend to counteract the effect of the lateral interactions (purple diamonds in Figure 4). These higher-order contributions are generally stabilizing for the coadsorption of CO but destabilizing for NO coadsorption. A stabilizing screening of the repulsive CO dipole interactions and a destabilizing quenching of the covalent bond of the NO dimer by the metal explain the counteracting behavior. This could be considered as “substrate mediation”.^17^ The compensation between lateral and three-body interactions accounts for the strong correlation between the combined adsorbate–substrate interactions and the adsorption energy.

Based on the many-body decomposition, we have shown that coadsorption at the same dopant is favored because of the stronger adsorption of both adsorbates at the dopant atom compared to the weaker adsorption on the host metal surfaces. This more than compensates for potentially repulsive lateral interactions, which, if present, are already largely offset by the higher-order three-body contributions.

### Optimal Number of Electrons Involved in Binding, As Suggested by the 10-Electron Count Rule, Explains the Particular Coadsorption Preference on Early TM Dopants

Similar to the trends observed across the periodic table for individual adsorption in atop position,^38^ we find a periodic pattern for adsorption energies for coadsorption at one site; see Figure S1 in the Supporting Information.

The trend for individual atop adsorption is attributed to an optimal value of 10 electrons for the sum of the electrons of the dopant element and the electrons of the adsorbate involved in binding.^38^ This rule aligns with the idea of free atom-like d states at the dopant atoms^28^ interacting with the near-frontier molecular orbitals^62^ and has been used to explain why adsorbates with more electrons bind more strongly to earlier transition metals. Coadsorbed systems follow this trend to some extent. When going from CO&CO (4 electrons), to CO&NO (5 electrons), to NO&NO (6 electrons),^38^ the dopant for which the binding is the strongest shifts to the left in the periodic table. However, the 10-electron count rule falls short of predicting the exact position of the minimum; see Figure S1 in the Supporting Information. In the coadsorption motif where the adsorbates are located at hollow sites, the adsorbate’s electrons can interact more strongly with the host. The overlap with the dopant orbitals is also changed and the local (orbital) symmetry is altered. This effectively reduces the number of adsorbate electrons interacting with the dopant.

Overall, when two adsorbates coadsorb at a dopant site, they collectively provide more electrons than one adsorbate individually adsorbed in the atop position. This surplus of electrons shifts the optimal TM element toward earlier TMs for a given adsorbate type. As a result, early TMs have a stronger preference for coadsorption compared to late TMs. This pattern also explains the enhanced binding of further CO molecules reported for the early 3d TMs V, Cr, and Mn,^26^ which aligns with our observations for the corresponding early 4d TMs Nb, Mo, and Tc; see the sequential interaction energies in the Tables S15 to S19 in the Supporting Information. The opposite trend is expected and observed for the late 3d TMs Fe, Co, and Ni in ref (26) and for the corresponding late 4d TMs Ru, Rh, and Pd in our work.

### Coadsorption Is Prevalent under Catalytic Conditions

**Figure 5:**
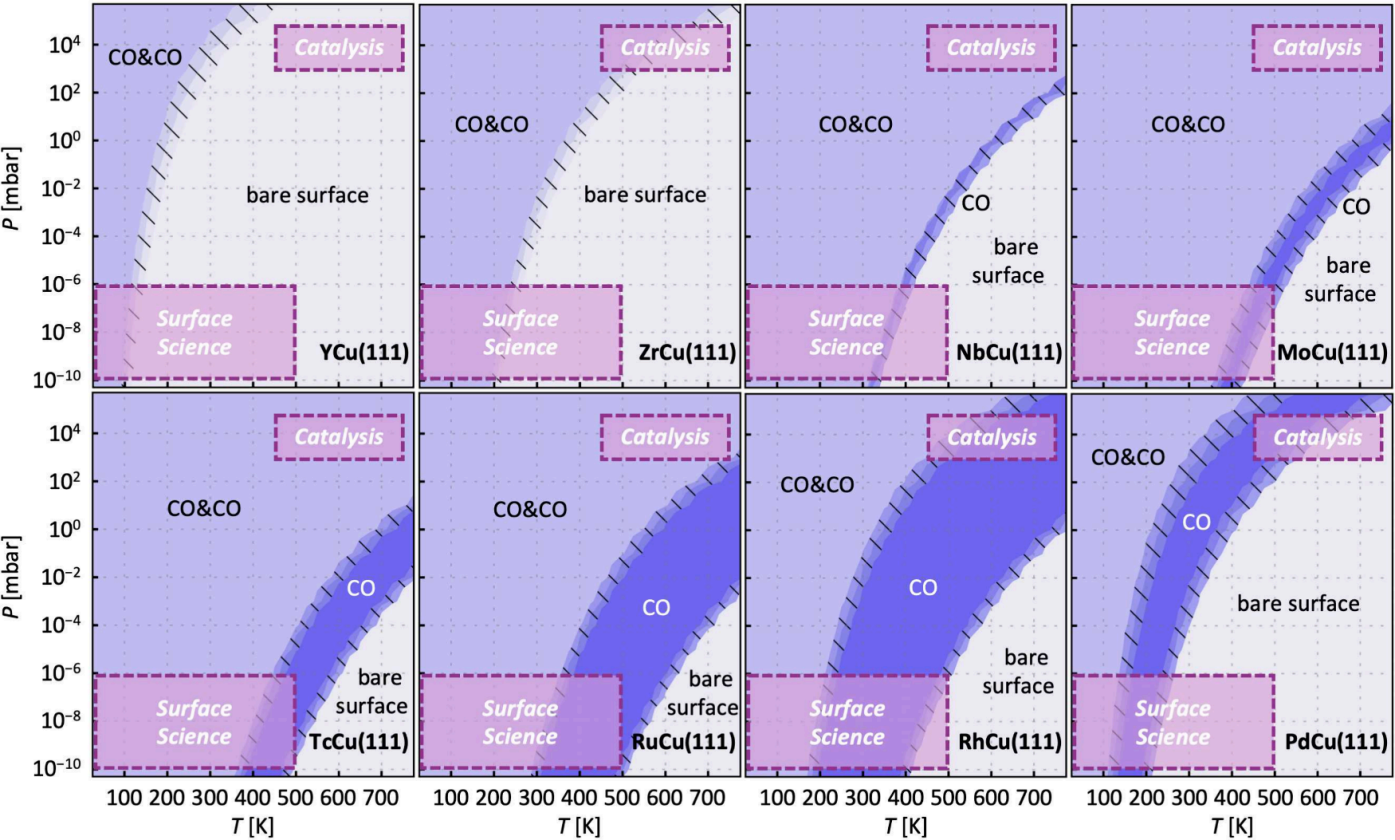
Pressure–temperature (P–T) phase diagrams show that CO coadsorption at a single dopant site is possible in both the surface science regime (very low pressures and lower temperatures) and the catalysis regime (high pressures and temperatures) in almost all single-atom alloys. The highlighted regimes “Surface Science” and “Catalysis” are meant to guide the eye and represent conditions commonly employed, but experiments may extend beyond these conditions. The phases considered are CO in the gas phase and a bare surface (light gray), CO individually adsorbed (dark purple), and CO coadsorbed at a dopant (light purple). Shaded areas represent 5 kJ mol^–1^ intervals to indicate regions of coexistence of two phases.

The preference for coadsorption at the same active site over individual adsorption is only one aspect that needs to be considered. In fact, to be relevant for catalytic applications, it is even more important that reactants adsorb from the gas phase onto a catalyst’s surface. Therefore, the question arises: At a given temperature and pressure, are adsorbates in the gas phase, individually adsorbed, or coadsorbed at an active site? Figure 5 shows the pressure–temperature (P–T) phase diagrams for CO adsorption on Cu-based SAAs containing 4d TM dopants. CO is chosen as adsorbate because it is a limiting case with strong repulsive lateral interactions. If coadsorption is observed for this molecule, other adsorbates that interact strongly with dopants will probably coadsorb as well.

In the high-pressure and high-temperature regime relevant for catalysis, as well as in the very low-pressure and low-temperature regime typically encountered in surface science experiments, coadsorption of CO at a dopant can indeed be thermodynamically favored on most SAAs. This is important for interpreting experimental observations from surface science as well as for understanding reaction mechanisms and designing computational studies under catalytic conditions. Given that SAAs have demonstrated the potential to lower reaction barriers and catalyze reactions under milder conditions compared to traditional metal surfaces, it is anticipated that the catalytic window will extend to lower temperatures, rendering the coadsorption motif even more significant.

For SAAs with a strong preference for coadsorption, such as NbCu(111), MoCu(111), and TcCu(111), the regime where individual CO adsorption is most stable (Figure 5, dark purple areas) is small or even vanishes on YCu(111) and ZrCu(111). For the SAAs with no (strong) preference for coadsorption, e.g. RhCu(111), individual CO adsorption is the most stable phase in a large P–T regime. As expected, the position of the coexistence line for the phase transition from gas-phase CO to adsorbed CO correlates with the strength of adsorption at the dopant site; see Figure S1 in the Supporting Information for adsorption energies. For weak CO adsorption, such as on YCu(111), ZrCu(111), and PdCu(111), the coexistence line at a given temperature is at higher pressures. For dopants that adsorb CO more strongly, such as MoCu(111), TcCu(111), and RuCu(111), the coexistence line with the gas phase is shifted to lower pressures.

### Catalytic Enhancement through Favorable Formation of Reaction-Ready States

The selection of adsorbates is also motivated by the promise coadsorption with hydrogen holds for reduction reactions. Both CO and NO reductions serve not only as useful model reactions but are also important chemical processes in their own right, as evidenced experimentally by the formation of C2 compounds^63,64^ or the conversion of the exhaust gas NO to N_2_^65^ on SAAs. The observed preference for coadsorption with hydrogen on early TM dopants aligns with experimental findings that the “single-atom Sc-doped Cu(111) surface exhibits excellent performance for C_2_H_4_ [formation]”.^64^*In light of these discoveries, we propose as a design strategy to utilize the so far little explored*^26,62,64,66−71^*early transition metals as dopants in SAAs to enhance catalytic performance for coupling reactions which are particularly difficult*^72^*with late transition metal dopants.*

The early TM dopants cause a stronger preference for coadsorption which could promote bimolecular and recombination reactions. Additionally, early TM-based SAAs typically form weaker bonds with the investigated adsorbates compared to late TM dopants, facilitating product desorption; see Section S2 and S3 in the Supporting Information for adsorption energies and preferences for coadsorption, respectively. However, it is important to note that reaction barriers for catalysts based on early TMs are usually comparatively high. This underscores the need for careful consideration of the specific early TM dopants depending on the intended reaction.

We demonstrate that enhancing catalytic activity in SAAs does not necessarily require lowering the reaction barriers for the rate-determining elementary step. Instead, this enhancement can be achieved through preferential coadsorption, which alters the overall reaction energy profile and affects the observed reaction rates by changing the most stable intermediate, even if the barrier for the elementary step of product formation remains unchanged. This aligns with the energetic span model, which identifies the most stable intermediate and the least stable transition state as key factors influencing reaction rates.^73,74^

**Figure 6:**
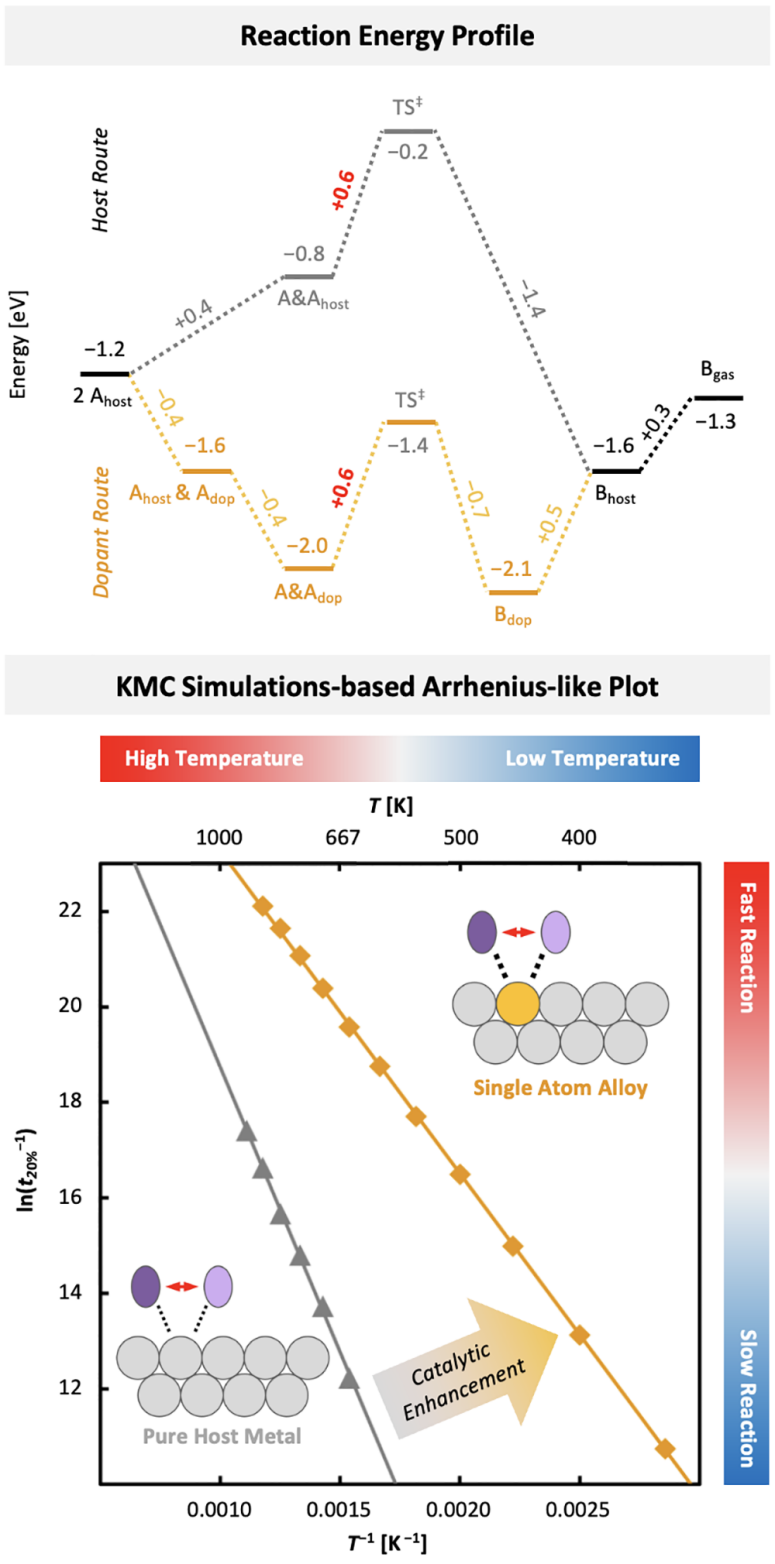
Top: Reaction energy profile for the dimerization reaction 2A → B on a host metal site (gray) and a dopant site as present in SAAs (orange), assuming the same barrier of 0.6 eV for the elementary reaction step for the formation of B from the coadsorbed state (red). The states considered for the kinetic Monte Carlo (KMC) simulations are the reactant A adsorbed on a host metal site, A_host_; the reactant A adsorbed on a dopant site, A_dop_; two reactants A coadsorbed on a host metal site, A&A_host_; two reactants A coadsorbed on a dopant site, A&A_dop_; the transition state, TS^‡^, for the formation of the product B from two reactants coadsorbed at the same site; the product B adsorbed on a host metal site, B_host_; the product B adsorbed on a dopant site, B_dop_; and the product B desorbed from the surface, B_gas_. Bottom: Arrhenius-like plot using the time, *t*_20%_, required until 20% of A initially adsorbed on the surface is converted to B desorbed from the surface, as obtained from KMC simulations at different temperatures, *T*, for the reaction on a pure metal (gray) and an SAA (orange) surface.

To further explore the effect of coadsorption preference on the overall reaction energy profile and thus catalytic enhancement, we develop two simple model reaction networks for the dimerization of two reactants, A, forming a product, B. One model represents a pure host metal surface, where coadsorption is energetically unfavorable, while the other represents a model SAA, where the reaction-ready coadsorption motif is energetically favored. We then perform KMC simulations to investigate the reaction rates for the formation of the product B and its desorption from the surfaces. Figure 6 shows Arrhenius-like plots based on KMC simulations conducted at different temperatures for these two model systems. Further details are available in Section S7 of the Supporting Information.

When coadsorption is favored, the reaction-ready coadsorption motif becomes the most energetically stable intermediate, meaning the barrier for the elementary step that forms product B primarily governs the reaction kinetics. In contrast, on a pure metal catalyst, the most stable state consists of two individually adsorbed reactants, A, and additional energy is required to bring them together. In this case, the overall reaction rate depends on both the energy needed to form the reaction-ready state and the barrier for the elementary step of product formation. This difference explains the simulated catalytic enhancement of 2 to 3 orders of magnitude at a given temperature and the lower overall activation energy observed when coadsorption is favored, as in the SAA model.

By assuming a most stable intermediate that is also the state required for product formation, the energy profile of the reaction changes, even if the barrier for the elementary step remains identical. We intentionally used the same barriers for the elementary steps in both energy profiles to specifically evaluate the effect of coadsorption, with all other factors being equal. In real systems, however, the reaction barriers at active dopant sites are likely lower, as dopants are selected to catalyze the reaction of interest more efficiently than the host metal.

KMC simulations naturally depend heavily on the underlying reaction energy profiles. These profiles, and consequently the reaction kinetics, vary based on the specific reaction and catalysts being investigated. The purpose of our simulations and analysis is to demonstrate that, while the barrier for the rate-determining elementary step plays a major role in influencing reaction kinetics, it is not the sole factor. The (de)stabilization of coadsorption states necessary for reactions also significantly shapes the overall reaction energy profile, thereby affecting the kinetics. Further, our analysis highlights that considering the most stable intermediates is crucial for understanding reaction mechanisms. Relying solely on the minima directly connected to the elementary reaction step for product formation may overlook key factors that influence the overall reaction kinetics.

### Coadsorption at the Same Dopant Can Induce a Redshift of CO and NO Stretching Vibration Frequencies

Experimental techniques such as scanning tunneling microscopy (STM) or IR spectroscopy can be used to detect signatures of coadsorption.^25^ On the one hand, such measurements serve to validate simulations. On the other hand, calculations can aid experimentalists in assigning peaks and identifying frequency shifts in spectra.

**Figure 7:**
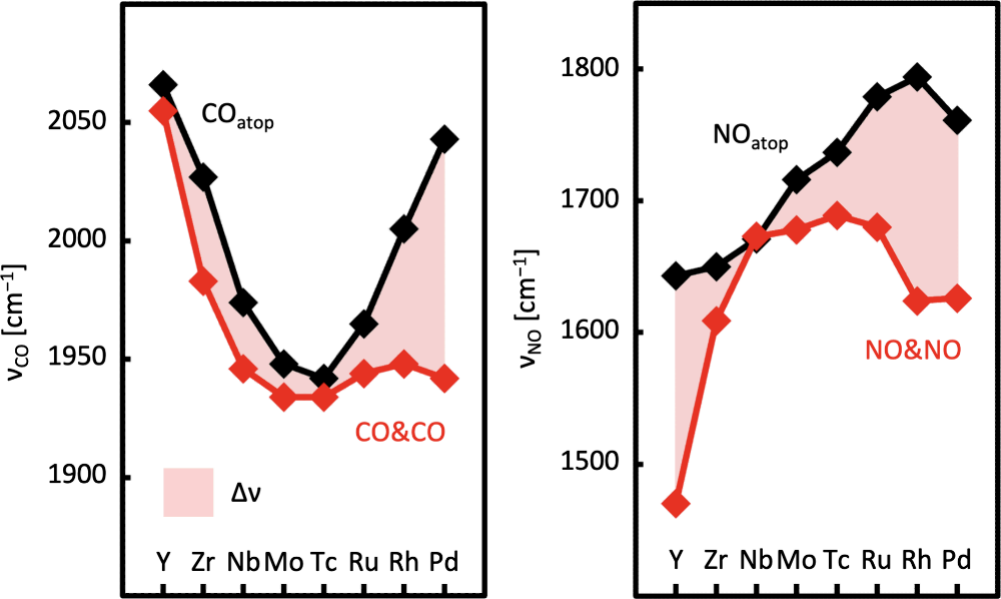
Scaled frequencies of the CO and NO stretching vibrations for separate adsorption in atop position (black) and of the symmetric stretching vibrations for CO&CO (left) and NO&NO (right) coadsorption at the same dopant site (red) on the Cu(111) surface. The red shaded area indicates the magnitude of the frequency shift, *Δν*, as calculated for the transition from the atop to the coadsorption motif. Connecting lines are for illustrative purposes only.

To assess the extent to which signatures of coadsorption can be observed with vibrational spectroscopy, we calculate (scaled) frequencies of the stretching vibrations for CO and NO separately adsorbed and coadsorbed at one dopant site on Cu(111). Periodic trends (red and black diamonds in Figure 7) as well as a tendency for a redshift that is strongest for very early and late and weakest for central TMs (redshaded area in Figure 7) are observed. Harmonic frequencies, scaled frequencies and the scaling approach as well as a comparison with experiments,^25,75−78^ which testifies good agreement, are provided in Section S6 of the Supporting Information.

Individual adsorption of CO and NO in the atop position of dopant sites induces a red shift in the vibrational frequencies of the intramolecular stretching vibrations compared to the experimental gas phase frequencies of 2134^79^ and 1876 cm^–1^,^80^ respectively. The trend for individual CO adsorption (black line in the left panel of Figure 7) indicates that the stronger the adsorption energy, the more pronounced the frequency redshift. This is expected due to the weakening of the C=O double bond upon adsorption.^81^ In contrast, the redshift predicted for individual NO adsorption in atop position (black line in the right panel of Figure 7) is strongest at early TMs and weakest at late TMs. Therefore, the redshift of NO is not correlated with the adsorption energies, which are strongest for central TM dopants, but may be explained by the greater ability of early TMs to donate electron density to NO. This electron density could populate antibonding π* orbitals, thus weakening the N=O bond.

For CO&CO and NO&NO coadsorption at the same dopant, we do indeed observe significant frequency shifts compared to individual atop adsorption, which are large enough to be detected experimentally. This is confirmed experimentally for CO coadsorption on RhCu, exhibiting a frequency shift of −52 cm^–1^,^25^ closely matching our predicted shift of −57 cm^–1^; see also Table S23 in the Supporting Information. Generally, the symmetric^25^ CO and NO stretching vibrational frequencies tend to shift further to the red upon coadsorption. However, the magnitude of this shift strongly varies across the periodic table (red shaded areas in Figure 7). It is largest for very early and especially late TM dopants, whereas it is smallest for central dopants. Therefore, solely relying on IR spectroscopy to detect coadsorption for central TMs might pose a challenge.

The trend in frequency shifts upon CO coadsorption at one site can be explained by two main factors: structural changes from atop adsorption to the coadsorption motif and alterations in the interaction of CO with the dopant. Coadsorbed molecules cannot both occupy atop positions on the same dopant but need to move to adjacent hollow sites. At these sites, back-donation from the d-states of the dopant and surrounding host metal atoms to the π* orbitals of CO is enhanced, leading to a redshift of the CO stretching vibration.^82−84^ Conversely, the interaction of CO with the dopant, and thus the dopant-induced redshift, decreases. For dopants strongly interacting with CO, such as central TMs, the attenuation of the dopant-induced redshift largely compensates the enhancement of back-donation due to structural changes. For dopants that interact less strongly with CO, such as late TMs, the weakening of interaction with CO results in a smaller change of the dopant-induced redshift. This manifests as a stronger net redshift as the redshift caused by structural changes cannot be compensated for by the reduced dopant-induced redshift.

## Conclusion

We have established that when the adsorbate coverage exceeds the number of accessible dopant sites, coadsorption at a dopant site becomes more favorable than separate adsorption. This is the important coverage regime for catalytic applications, considering the low concentration of dopant atoms in SAAs and the high reaction pressures usually employed for catalysis. Additionally, phase diagrams show that this coadsorption motif is indeed the most stable phase for several SAAs under temperature and pressure conditions commonly encountered in surface science experiments and catalytic processes.

The preference for coadsorption at the same dopant is a general phenomenon, as demonstrated for three adsorbates (CO, NO, H), 13 transition metal dopants (3d: Ni, Cu; 4d: Y, Zr, Nb, Mo, Tc, Ru, Rh, Pd, Ag; 5d: Ir, Pt), as well as for two terrace site host surfaces (Cu(111), Ag(111)) and the Cu(211) surface, which serves as a model for step edge defects. This preference is consistent across various DFT functionals and is also expected to hold for larger adsorbates as long as repulsive effects, such as steric hindrance, between the adsorbates do not outweigh the energetic stabilization due to their interaction with the dopant site.

Coadsorption at one site can induce a redshift in the intramolecular stretching vibration frequency of CO and NO. While this redshift tends to be more pronounced for very early and in particular late TM dopants, it is relatively small for central TMs. Nonetheless, combining calculations and experiments will help to identify and assign spectroscopic signatures of coadsorption motifs in experimental samples.

The periodic trends in adsorption energies observed for coadsorption can be partially explained by the 10-electron count rule,^38^ which has been proposed for single adsorption in atop position. Although this rule cannot directly be applied to coadsorption, assuming an optimal electron count explains the pronounced preference for coadsorption on early TM dopants.

Our KMC simulations reveal that the catalytic activity of SAAs can be enhanced compared to pure metal surfaces not only due to changes in the barriers of the rate-determining elementary reaction steps but also through preferential coadsorption. Changes in coadsorption preference can alter the most stable intermediate, thereby affecting the overall reaction energy profile and, consequently, the reaction kinetics. This enhancement arises because, in systems with favorable coadsorption, only the barrier for the elementary step needs to be overcome, whereas in systems in which coadsorption is destabilized, both the energy to form the reaction-ready state and the barrier for the elementary reaction contribute to the overall reaction barrier. This illustrates the critical role of coadsorption at one active site in understanding kinetics and performance of such catalysts.

Finally, our findings suggest that further investigations into early TM-containing SAAs are worthwhile. These systems exhibit a strong preference for coadsorption alongside comparatively weak adsorption. This exceptional combination of catalyst properties could stabilize the coadsorbed state of bimolecular and recombination reactions at an active site while facilitating product desorption. Investigating the adsorbate-induced response of the active sites^85,86^ of such catalysts or segregation^87^ and aggregation^71^ tendencies are therefore interesting research avenues for future studies.

Moreover, coadsorption of CO and NO with the reducing agent hydrogen at the same dopant is not only favored for most TMs if no further dopant sites are accessible, but even if vacant dopant atoms are still available in the case where early TMs are used as dopants. This may hint to new routes for more efficient reduction reactions under milder conditions.

## Supporting Information Available

Computational details, adsorption energies, preferences for coadsorption, dependency on DFT functional, many-body decomposition of adsorption energy, setups for the kinetic Monte Carlo simulations, and vibrational frequencies are reported (PDF)
Optimized structures and example input files (ZIP)
