# Supplementary material for: Bringing Molecules Together: Synergistic Coadsorption at Dopant Sites of Single Atom Alloys
Source: J Am Chem Soc. 2024 Oct 2;146(41):28119–30. doi: 10.1021/jacs.4c07621 (PMC11487606; doi:10.1021/jacs.4c07621)
Supplement: Supplementary file 1 — ja4c07621_si_001.pdf [file ja4c07621_si_001.pdf]

Supporting Information: Bringing Molecules Together: Synergistic Coadsorption at Dopant Sites of Single Atom Alloys

Fabian Berger,[*,†] Julia Schumann,[†,‡] Romain Réocreux,[†,‡] Michail Stamatakis,[‡,¶] and Angelos Michaelides[*,†]

†*Yusuf Hamied Department of Chemistry, University of Cambridge, CB2 1EW Cambridge, UK*

‡*Thomas Young Centre and Department of Chemical Engineering, University College London, WC1E 7JE London, UK*

¶*Department of Chemistry, University of Oxford, OX1 3QZ Oxford, UK*

E-mail: fb593@cam.ac.uk; am452@cam.ac.uk

Contents

S1 Computational Details **S-3**

S2 Adsorption Energies **S-5**

S3 Preferences for Coadsorption at the same Site **S-12**

S4 Dependence of Adsorption Energies and Preferences for Coadsorption on the DFT Functional **S-18**

S5 Many-body Decomposition of the Adsorption Energy **S-23**

S6 Vibrational Frequencies **S-37**

S7 Kinetic Monte Carlo Simulations **S-42**

References **S-46**

S1 Computational Details

The metal surfaces are modeled with a 3×3×5 supercell and truncated at the (111) and (211) facet, with 15 Å vacuum height, and two fixed bottom layers. Periodic density functional theory (DFT) calculations with the revised Perdew Burke Ernzerhof (RPBE)[S1] functional are performed as implemented in VASP 6.3.0, using plane wave basis sets for valence electrons and the projector augmented wave (PAW) method for core electrons with standard potentials.[S2–S5] Further, spin-polarization, first order Methfessel-Paxton smearing (SIGMA = 0.1), dipole correction, and minimum valency pseudopotentials are used. The same settings are used to test the effects of using different functionals (PBE,[S6] PBEsol,[S7,S8] optB86b-vdW[S9]).

For structure (atom position) optimizations, the energy cutoff is set to 400 eV and the Brillouin zone is sampled using a Monkhorst-Pack mesh with 13×13×1 **k** points. The convergence thresholds were set to 10^{-6} eV for electronic energy changes and to 0.01 eV $Å^{-1}$ for structural changes. Harmonic partial Hessians are calculated numerically using central finite differences with Cartesian displacements of 1 pm and an energy convergence threshold of 10^{-7} eV to calculate vibrational frequencies. For the partial Hessians, only the adsorbates and the dopant atom are allowed to move. Example inputs are provided as separate files.

Unit cell vectors are determined using the equation of state method[S10] as implemented in the Atomic Simulation Environment (ASE), an energy cutoff of 800 eV is used to minimize the influence of volume change on the basis set, and the Brillouin zone is sampled using a Monkhorst-Pack mesh with 31×31×31 **k** points.

Pressure-temperature phase diagrams are obtained using the ASE thermochemistry class to calculate the Gibbs free energies of adsorption for three phases: (1) CO in the gas phase on a bare surface, (2) CO individually adsorbed at a dopant atom, and (3) two CO molecules coadsorbed at the same dopant site. Vibrational, thermal, and entropy contributions are incorporated by using the calculated vibrational frequencies for CO molecules, which account for the internal stretching vibrations as well as hindered rotations and translations.

Kinetic Monte Carlo (KMC) simulations are performed using the graph-theoretical KMC

framework as implemented in Zacros version 3.02.[S11–S14] The simulated surfaces consist of (50 × 50) rectangular unit cells, resulting in 2500 adsorption sites per periodic simulation cell. For the SAA model, 25 dopant sites randomly replace host sites, mimicking a dopant concentration of 1%. Each site is connected to four neighboring sites. The initial coverage is set to 15%, corresponding to 375 randomly placed species A. Further details about the model are provided in Section S7.

S2 Adsorption Energies

The reference states for individual H atoms generally favor adsorption at hollow fcc sites, with the exception of the investigated 5d dopants, which cause a preference for the atop position. This is consistent with previous observations.[S15] For individual NO adsorption, the atop position at the dopant is generally favored. In agreement with the literature,[S16,S17] H and NO preferentially adsorb at hollow fcc sites on pure Cu(111) and Ag(111) surfaces. The adsorption of CO occurs in the atop position on pure Cu(111) and Ag(111) surfaces[S18] and is thus expected to behave similarly on dopant sites of SAAs. However, it is worth noting that predicting the correct site preference for CO adsorption on TM surfaces is a notorious problem for density functional theory (DFT), known as the ‘CO/Pt(111) Puzzle’.[S19]

Table S1: Adsorption energies, E_{ads}, for the individual adsorption of CO, NO, and H as well as coadsorption of CO&CO, CO&NO, NO&NO, H&H, H&H&H, CO&H, and NO&H at the same site in kJ mol^{-1} on the Cu(111) surface. The atop position, H_{top} and NO_{top}, and the hollow fcc site, H_{fcc} and NO_{fcc}, are considered for the adsorption of H and NO, respectively. A "/" indicates an unstable or unconsidered configuration. "Cu_{fcc}" and "atop" describe the final adsorption motif.

TM	H_{top}	H_{fcc}	H&H	H&H&H	CO	NO_{top}	NO_{fcc}	CO&CO	NO&NO	CO&NO	CO&H	NO&H
Ni	–212	–248	–497	–737	–139	–156	–144	–196	–257	–229	–366	–387
Cu	–174	–230	–461	–682	–48	–48	–84	/	/	/	–281	–310
Y	–183	–237	–473	–710	–45	–148	atop	–112	–270	–193	–298	–385
Zr	–212	–247	–498	–744	–88	–194	–205	–199	–378	–295	–340	–444
Nb	–217	–255	–511	–774	–141	–269	atop	–279	–467	–379	–404	–520
Mo	–221	–257	–516	–790	–179	–310	atop	–335	–507	–430	–446	–561
Tc	–231	–257	–533	–807	–205	–323	atop	–364	–490	–433	–471	–567
Ru	–241	–260	–535	–807	–214	–290	atop	–341	–395	–373	–467	–516
Rh	–230	–250	–501	–755	–172	–190	–151	–251	–269	–259	–401	–404
Pd	–195	–232	–463	–688	–88	–63	–88	–129	–153	–138	–304	–316
Ag	–145	–219	–437	–651	–2	–3	Cu_{fcc}	/	/	/	–261	–302
Ir	–262	–258	–530	–791	–205	–217	atop	–277	–273	–276	–435	–418
Pt	–234	–236	–471	–699	–120	–79	–86	–139	–153	–141	–321	–317

Table S2: Adsorption energies, E_{ads}, for the individual adsorption of CO, NO, and H as well as coadsorption of CO&CO, CO&NO, NO&NO, H&H, H&H&H, CO&H, and NO&H at the same site in kJ mol^{-1} **per adsorbate** on the Cu(111) surface. The atop position, H_{top} and NO_{top}, and the hollow fcc site, H_{fcc} and NO_{fcc}, are considered for the adsorption of H and NO, respectively. A "/" indicates an unstable or unconsidered configuration. "Cu_{fcc}" and "atop" describe the final adsorption motif.

TM	H_{top}	H_{fcc}	H&H	H&H&H	CO	NO_{top}	NO_{fcc}	CO&CO	NO&NO	CO&NO	CO&H	NO&H
Ni	–212	–248	–248	–246	–139	–156	–144	–98	–128	–115	–183	–194
Cu	–174	–230	–230	–227	–48	–48	–84	/	/	/	–140	–155
Y	–183	–237	–237	–237	–45	–148	atop	–56	–135	–96	–149	–193
Zr	–212	–247	–249	–248	–88	–194	–205	–100	–189	–147	–170	–222
Nb	–217	–255	–256	–258	–141	–269	atop	–140	–234	–189	–202	–260
Mo	–221	–257	–258	–263	–179	–310	atop	–167	–253	–215	–223	–280
Tc	–231	–257	–267	–269	–205	–323	atop	–182	–245	–217	–236	–284
Ru	–241	–260	–267	–269	–214	–290	atop	–170	–197	–186	–233	–258
Rh	–230	–250	–250	–252	–172	–190	–151	–125	–135	–130	–201	–202
Pd	–195	–232	–232	–229	–88	–63	–88	–65	–77	–69	–152	–158
Ag	–145	–219	–218	–217	–2	–3	Cu_{fcc}	/	/	/	–130	–151
Ir	–262	–258	–265	–264	–205	–217	atop	–138	–136	–138	–217	–209
Pt	–234	–236	–235	–233	–120	–79	–86	–69	–76	–70	–161	–158

Table S3: Adsorption energies, E_{ads}, for the individual adsorption of CO and H as well as coadsorption of CO&CO and CO&H at the same site in kJ mol^{-1} on the Cu(211) surface. The bridge, H_{bridge}, and hollow site, H_{hollow}, are considered. A "/" indicates an unstable or unconsidered configuration.

TM	H_{bridge}	H_{hollow}	CO	CO&CO	CO&H
Cu	/	–232	–67	/	/
Y	–260	/	/	–150	–339
Zr	–278	/	–118	–239	–386
Nb	–277	/	–142	–309	–426
Mo	–267	/	–158	–343	–445
Tc	–266	/	–189	–384	–467
Ru	–267	/	–211	–377	–479
Rh	–254	/	–180	–299	–419
Pd	/	–232	–99	–171	–326

Table S4: Adsorption energies, E_{ads}, for the individual adsorption of CO and H as well as coadsorption of CO&CO and CO&H at the same site in kJ mol^{-1} **per adsorbate** on the Cu(211) surface. The bridge, H_{bridge}, and hollow site, H_{hollow}, are considered. A "/" indicates an unstable or unconsidered configuration.

TM	H_{bridge}	H_{hollow}	CO	CO&CO	CO&H
Cu	/	–232	–67	/	/
Y	–260	/	/	–75	–169
Zr	–278	/	–118	–120	–193
Nb	–277	/	–142	–155	–213
Mo	–267	/	–158	–171	–222
Tc	–266	/	–189	–192	–234
Ru	–267	/	–211	–188	–240
Rh	–254	/	–180	–149	–209
Pd	/	–232	–99	–86	–163

Table S5: Adsorption energies, E_{ads}, for the individual adsorption of CO, NO, and H as well as coadsorption of CO&CO, CO&NO, NO&NO, H&H, H&H&H, CO&H, and NO&H at the same site in kJ mol^{-1} on the Ag(111) surface. The atop position, H_{top} and NO_{top}, and the hollow fcc site, H_{fcc} and NO_{fcc}, are considered for the adsorption of H and NO, respectively. A "/" indicates an unstable or unconsidered configuration. "atop" describes the final adsorption motif.

TM	H_{top}	H_{fcc}	H&H	H&H&H	CO	NO_{top}	NO_{fcc}	CO&CO	NO&NO	CO&NO	CO&H	NO&H
Ni	–216	–232	–462	–682	–153	–176	atop	–185	–236	–198	–354	–375
Cu	–177	–205	–411	–607	–50	–55	–56	/	/	/	–235	–250
Y	–187	–223	–446	–666	–56	–157	atop	–123	–291	–208	–281	–371
Zr	–219	–241	–479	–719	–118	–245	atop	–224	–434	–335	–356	–472
Nb	–219	–242	–485	–732	–154	–305	atop	–293	–512	–412	–401	–539
Mo	–221	–239	–482	–733	–157	–305	atop	–320	–512	–425	–411	–543
Tc	–230	–242	–496	–775	–202	–327	atop	–371	–512	–445	–456	–570
Ru	–249	–255	–531	–812	–237	–323	atop	–373	–439	–409	–487	–547
Rh	–241	–247	–502	–749	–197	–222	atop	–265	–266	–272	–419	–427
Pd	–197	–217	–431	–636	–98	–72	atop	–95	–118	–95	–287	–283
Ag	–146	–192	–383	–569	0	–7	–18	/	/	/	–194	–205
Ir	–277	atop	–551	–815	–238	–254	atop	–312	–285	–307	–468	–458
Pt	–241	atop	–471	–683	–139	–92	atop	–128	–141	–118	–328	–312

Table S6: Adsorption energies, E_{ads}, for the individual adsorption of CO, NO, and H as well as coadsorption of CO&CO, CO&NO, NO&NO, H&H, H&H&H, CO&H, and NO&H at the same site in kJ mol^{-1} **per adsorbate** on the Ag(111) surface. The atop position, H_{top} and NO_{top}, and the hollow fcc site, H_{fcc} and NO_{fcc}, are considered for the adsorption of H and NO, respectively. A "/" indicates an unstable or unconsidered configuration. "atop" describes the final adsorption motif.

TM	H_{top}	H_{fcc}	H&H	H&H&H	CO	NO_{top}	NO_{fcc}	CO&CO	NO&NO	CO&NO	CO&H	NO&H
Ni	–216	–232	–231	–227	–153	–176	atop	–92	–118	–99	–177	–188
Cu	–177	–205	–206	–202	–50	–55	–56	/	/	/	–117	–125
Y	–187	–223	–223	–222	–56	–157	atop	–62	–145	–104	–140	–185
Zr	–219	–241	–240	–240	–118	–245	atop	–112	–217	–167	–178	–236
Nb	–219	–242	–242	–244	–154	–305	atop	–147	–256	–206	–201	–269
Mo	–221	–239	–241	–244	–157	–305	atop	–160	–256	–213	–205	–271
Tc	–230	–242	–248	–258	–202	–327	atop	–185	–256	–222	–228	–285
Ru	–249	–255	–266	–271	–237	–323	atop	–187	–219	–205	–244	–274
Rh	–241	–247	–251	–250	–197	–222	atop	–133	–133	–136	–209	–214
Pd	–197	–217	–215	–212	–98	–72	atop	–47	–59	–47	–144	–141
Ag	–146	–192	–192	–190	0	–7	–18	/	/	/	–97	–103
Ir	–277	atop	–275	–272	–238	–254	atop	–156	–143	–154	–234	–229
Pt	–241	atop	–236	–228	–139	–92	atop	–64	–71	–59	–164	–156

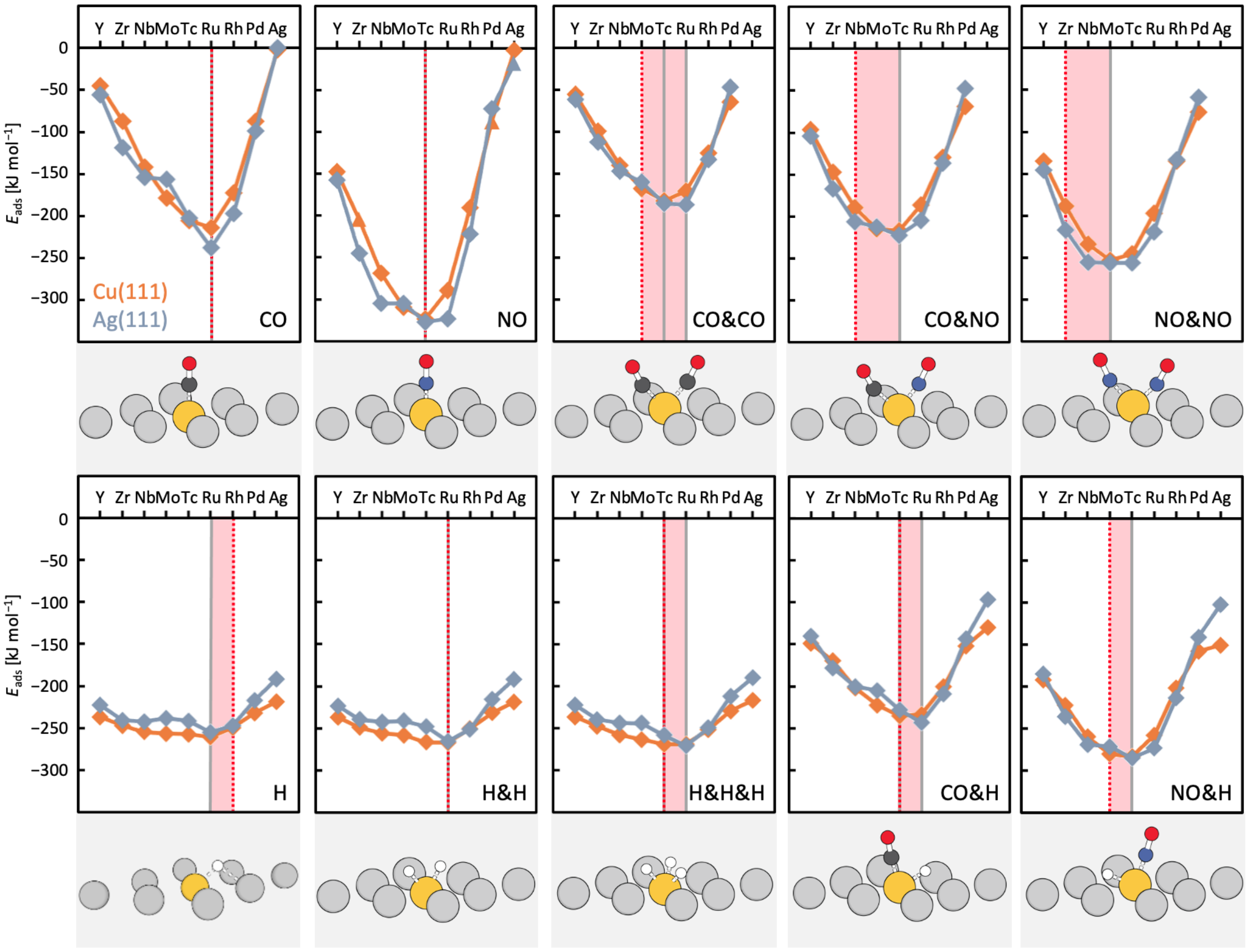

Figure S1: Adsorption energies, E_{ads}, for the individual adsorption of CO, NO, and H as well as coadsorption of CO&CO, CO&NO, NO&NO, H&H, H&H&H, CO&H, and NO&H at the same site in kJ mol^{-1} per adsorbate on Cu(111) (orange) and Ag(111) (blue) based SAAs, see Eq. 1 in the main text. The gas phase references are CO and NO molecules as well as the H atom. The dotted red lines indicate the TM dopant element predicted to be the most stable according to the 10-electron count rule,[S20] the solid gray lines mark the dopant elements calculated to be the most stable, and the red shaded areas highlight the discrepancy between them. Beneath each panel an example of an adsorption structure on the Ag(111) surface is shown. Symbols: CO_{atop}, NO_{atop}, H_{fcc}, CO_{host}&CO_{dop}, NO_{host}&NO_{dop}, CO_{host}&NO_{dop}, H_{host}&CO_{dop}, and H_{host}&NO_{dop} – diamonds; NO_{fcc} – triangles. Color code: dopant element – yellow, Ag – gray, C – black, H – white, N – blue, O – red.

S3 Preferences for Coadsorption at the same Site

Table S7: Preference for coadsorption of H&H, CO&CO, NO&NO, CO&NO, CO&H, and NO&H at the same site in kJ mol^{-1} per adsorbate on the Cu(111) surface (see Eq. 3 in the main text; difference of the adsorption energies between coadsorption at the same dopant site and both adsorbates individually adsorbed at a dopant site). Unless otherwise stated, the hollow fcc site is the most stable and is therefore used for the individual adsorption of H, whereas the atop site is used for the individual adsorption of CO and NO. A "/" indicates an unstable or unconsidered configuration.

TM	H&H	H&H&H	CO&CO	NO&NO	CO&NO	CO&H	NO&H
Ni	0	3	41	28	33	11	9
Cu	0	3	/	/	/	–1	2[a]
Y	0	0	–11	13	0	–8	0
Zr	–2	–1	–12	16[a]	–1[a]	–2	4[a]
Nb	–1	–3	2	35	16	–4	2
Mo	–1	–7	11	56	30	–5	3
Tc	–9	–12	23	78	48	–4	7
Ru	–7	–9	43	92	65	4	17
Rh	–1	–2	47	56	52	10	18
Pd	0	3	23	11[a]	19[a]	8	2[a]
Ag	0	2	/	/	/	–20	–40
Ir	–3[b]	–1[b]	67	81	73	16[b]	31[b]
Pt	0	3	51	10[a]	33[a]	18	2[a]

[a] Individual NO at hollow fcc site

[b] Individual H at atop site

Table S8: Preference for coadsorption of CO&CO and CO&H at the same site in kJ mol^{-1} per adsorbate on the Cu(211) surface (see Eq. 3 in the main text; difference of the adsorption energies between coadsorption at the same dopant site and both adsorbates individually adsorbed at a dopant site). Unless otherwise stated, the bridge site is the most stable and is therefore used for the individual adsorption of H, whereas the atop site is used for the individual adsorption of CO. A "/" indicates an unstable or unconsidered configuration.

TM	CO&CO	CO&H
Y	/	/
Zr	–2	5
Nb	–12	–3
Mo	–14	–10
Tc	–3	–6
Ru	23	0
Rh	30	7
Pd	14	3[a]

[a] Individual H at hollow site; $CO\&H_{hollow}$

Table S9: Preference for coadsorption of H&H, CO&CO, NO&NO, CO&NO, CO&H, and NO&H at the same site in kJ mol^{-1} per adsorbate on the Ag(111) surface (see Eq. 3 in the main text; difference of the adsorption energies between coadsorption at the same dopant site and both adsorbates individually adsorbed at a dopant site). Unless otherwise stated, the hollow fcc site is the most stable and is therefore used for the individual adsorption of H, whereas the atop site is used for the individual adsorption of CO and NO. A "/" indicates an unstable or unconsidered configuration.

TM	H&H	H&H&H	CO&CO	NO&NO	CO&NO	CO&H	NO&H
Ni	1	5	61	58	66	15	16
Cu	0	3	/	/	/	11	6[a]
Y	0	1	–6	12	3	–1	5
Zr	1	1	7	28	14	1	7
Nb	0	–2	8	49	24	–2	4
Mo	–3	–6	–3	49	18	–8	0
Tc	–6	–17	17	71	42	–6	0
Ru	–10	–15	51	104	75	3	16
Rh	–4	–3	64	89	73	13	21
Pd	2	5	51	13	38	14	3
Ag	0	2	/	/	/	–1	2[a]
Ir	1[b]	5[b]	82	112	93	23[b]	36[b]
Pt	5[b]	13[b]	75	22	57	26[b]	11[b]

[a] Individual NO at hollow fcc site

[b] Individual H at atop site

Table S10: Preference for coadsorption of H&H, CO&CO, NO&NO, CO&NO, CO&H, and NO&H at the same site in kJ mol^{-1} per adsorbate on the Cu(111) surface (see Eq. 2 in the main text; difference of the adsorption energies between coadsorption at the dopant site and the most stable combination of individual adsorption at the dopant site accompanied by individual adsorption of the other adsorbate on the pure metal surface). Unless otherwise stated, the hollow fcc site is used for the individual adsorption of H and NO_{host}, whereas the atop site is used for the individual adsorption of CO and NO_{dop}. A "/" indicates an unstable or unconsidered configuration.

TM	H&H	CO&CO	NO&NO	CO&NO	CO&H	NO&H
Ni	–9	–4	–8	–3[b]	1[b]	0[a]
Cu	0	/	/	/	–1	2[c]
Y	–3	–9	–19	1[a]	–6[b]	–4[a]
Zr	–10	–32	–44[c]	–21[ac]	–11[a]	–5[ac]
Nb	–13	–45	–57	–31[a]	–16[a]	–11[a]
Mo	–15	–54	–57	–36[a]	–18[a]	–10[a]
Tc	–23	–55	–41	–31[a]	–18[a]	–7[a]
Ru	–22	–40	–11	–18[a]	–12[a]	2[a]
Rh	–11	–15	3	–1[b]	0[a]	8[a]
Pd	–1	3	9[c]	17[b]	7[a]	1[ac]
Ag	6	/	/	/	3[b]	0[b]
Ir	–19[d]	–12	14	6[b]	0[a]	15[a]
Pt	–2	15	9[c]	32[b]	15[a]	1[b]

[a] NO_{dop}&CO_{host}, CO_{dop}&H_{host}, NO_{dop}&H_{host}

[b] CO_{dop}&NO_{host}, H_{dop}&CO_{host}, H_{dop}&NO_{host}

[c] Individual NO at dopant hollow fcc site

[d] Individual H at dopant atop site

Table S11: Preference for coadsorption of CO&CO and CO&H at the same site in kJ mol^{-1} per adsorbate on the Cu(211) surface (see Eq. 2 in the main text; difference of the adsorption energies between coadsorption at the dopant site and the most stable combination of individual adsorption at the dopant site accompanied by individual adsorption of the other adsorbate on the pure metal surface). Unless otherwise stated, the bridge site is the most stable and is therefore used for the individual adsorption of H, whereas the atop site is used for the individual adsorption of CO. A "/" indicates an unstable or unconsidered configuration.

TM	CO&CO	CO&H
Y	/	–6[a]
Zr	–27	–21[a]
Nb	–50	–26
Mo	–59	–27
Tc	–64	–23
Ru	–49	–18
Rh	–26	–3
Pd	–3	3

[a] CO_{host}&H_{dop}

Table S12: Preference for coadsorption of H&H, CO&CO, NO&NO, CO&NO, CO&H, and NO&H at the same site in kJ mol^{-1} per adsorbate on the Ag(111) surface (see Eq. 2 in the main text; difference of the adsorption energies between coadsorption at the dopant site and the most stable combination of individual adsorption at the dopant site accompanied by individual adsorption of the other adsorbate on the pure metal surface). Unless otherwise stated, the hollow fcc site is used for the individual adsorption of H and NO_{host}, whereas the atop site is used for the individual adsorption of CO and NO_{dop}. A "/" indicates an unstable or unconsidered configuration.

TM	H&H	CO&CO	NO&NO	CO&NO	CO&H	NO&H
Ni	–19	–16	–21	–11[a]	–5[a]	–4[a]
Cu	–7	/	/	/	4[a]	–1[ac]
Y	–16	–34	–57	–25[a]	–16[a]	–11[a]
Zr	–24	–53	–86	–45[a]	–23[a]	–18[a]
Nb	–25	–70	–94	–54[a]	–28[a]	–21[a]
Mo	–26	–82	–95	–60[a]	–31[a]	–23[a]
Tc	–31	–84	–83	–59[a]	–31[a]	–25[a]
Ru	–42	–68	–49	–43[a]	–29[a]	–16[a]
Rh	–32	–34	–13	–25[a]	–15[a]	–7[a]
Pd	–11	2	–13	11[b]	2[a]	–9[a]
Ag	0	/	/	/	–1	2[c]
Ir	–41[d]	–37	–6	–25[b]	–19[a]	–6[a]
Pt	–19[d]	5	–15	20[b]	1[a]	–14[a]

[a] NO_{dop}&CO_{host}, CO_{dop}&H_{host}, NO_{dop}&H_{host}

[b] CO_{dop}&NO_{host}, H_{dop}&CO_{host}, H_{dop}&NO_{host}

[c] Individual NO at dopant hollow fcc site

[d] Individual H at dopant atop site

S4 Dependence of Adsorption Energies and Preferences for Coadsorption on the DFT Functional

The adsorption energies are strongly affected by the DFT functional choice. We investigate the effect of the commonly used exchange-correlation potentials RPB,[S1] PBE,[S6] and PBEsol,[S7,S8] as well as the functional optB86b-vdW,[S9] which incorporates non-local correlation that accounts for dispersion interactions. These functionals yield significantly different adsorption energies. RPBE predicts the weakest adsorption of CO and NO, and PBE, PBEsol, as well as optB86b-vdW overestimate adsorption by an average of 17, 42, and 37 kJ mol^{-1}, respectively, compared to RPBE for a selected subset of the systems, see Figure S2 and Table S13. Since, the average standard deviation for the coadsorption preference is only 3 kJ mol^{-1}, see Figure S2 and Table S14, we expect the RPBE functional to predict the correct trends. This functional is also known to accurately describe CO and NO adsorption on TM surfaces.[S21]

We further investigated the performance of the foundation model MACE-MP-0,[S22] which is a machine learning potential (MLP) based on the MACE architecture[S23] and developed to reproduce the DFT functional PBE. It shows good accuracy for the dopant elements Zr, Tc, Ru, Rh and Pd, but significantly underperforms for the dopant element Mo.

This discrepancy can be attributed to the rules of the Materials Project (MP),[S24] which were used to generate the training data for MACE-MP-0. The MP requires a Hubbard U potential to be applied to systems containing the elements Co, Cr, Fe, Mn, Mo, Ni, V or W in combination with oxygen atoms. While this rule reflects the original intent of the MP, when it comes to transition metal oxides, it does cause unintended inconsistencies in the description of oxygen-containing adsorbates such as CO or NO that interact with metal surfaces containing any of the aforementioned transition elements. Since MACE-MP-0 relies on locality, the energies and gradients it predicts resemble DFT+U results rather than pure DFT results when an oxygen atom is in the vicinity of one of these transition metal elements.

This discrepancy may explain why the adsorption energies of MACE-MP-0 for systems with CO, but not H, differ significantly from their PBE references, see Figure S2 as well as Tables S13 and S14. However, for metallic systems, no (implicit) Hubbard U potential should be applied to these transition metal atoms when an oxygen atom is nearby, which may indicate a general limitation of the current version of the foundation model.

Table S13: Adsorption energies, E_{ads}, for the individual adsorption of CO and H in atop position and the coadsorption of CO&CO and CO&H at the same site on the Cu(111) surface, in kJ mol^{-1} per adsorbate, as determined with the DFT functionals RPBE, PBE, PBEsol and optB86b-vdW and with the foundation model MACE-MP-0. The standard deviations, σ, only take into account the DFT results. A "/" indicates an unstable or unconsidered configuration.

		Cu	Y	Zr	Nb	Mo	Tc	Ru	Rh	Pd
$E_{\text{ads}}(\text{H}_{\text{top}})$	RPBE	–174	–183	–212	–217	–221	–231	–241	–230	–195
	PBE	–183	–174	–210	–221	–229	–241	–250	–240	–205
	PBEsol	–201	–166	–208	–228	–244	–260	–267	–259	–226
	optB86b-vdW	–208	–197	–235	–247	–256	–268	–273	–264	–230
	MACE-MP-0	–172	–167	–212	–251	–238	–286	–258	–243	–184
	σ	14	11	11	11	14	15	13	14	14
$E_{\text{ads}}(\text{CO})$	RPBE	–48	–45	–88	–141	–179	–205	–214	–172	–88
	PBE	–71	–54	–103	–161	–206	–231	–237	–196	–111
	PBEsol	-99	–60	–117	–182	–235	–263	–267	–227	–141
	optB86b-vdW	–87	–66	–118	–176	–224	–248	–251	–210	–125
	MACE-MP-0	–109	–77	–69	–125	–99	–241	–247	–213	–84
	σ	19	8	12	16	21	21	20	20	20
$E_{\text{ads}}(\text{CO\&CO})$	RPBE	/	–56	–100	–140	–167	–182	–170	–125	–65
	PBE	/	–70	–122	–164	–196	–210	–198	–154	–94
	PBEsol	/	/	–148	–193	–229	–246	–234	–191	–131
	optB86b-vdW	/	–87	–142	–183	–215	–229	–216	–172	–113
	MACE-MP-0	/	–137	–129	–150	–119	–235	–228	–193	–126
	σ	/	13	19	20	23	24	23	24	25
$E_{\text{ads}}(\text{CO\&H})$	RPBE	–140	–149	–170	–202	–223	–236	–233	–201	–152
	PBE	–163	–164	–183	–217	–242	–255	–252	–219	–174
	PBEsol	–194	/	–209	–239	–268	–282	–278	–247	–206
	optB86b-vdW	–183	–184	–206	–236	–262	–274	–269	–237	–194
	MACE-MP-0	–193	–222	–203	–209	–192	–260	–258	–237	–188
	σ	21	15	16	15	18	18	17	18	20

Table S14: Preference for coadsorption, ΔE_{coads}, of CO&CO and CO&H at the same site in kJ mol^{-1} per adsorbate on the Cu(111) surface (see Eq. 3 in the main text; difference of the adsorption energies between coadsorption at the dopant site and individual adsorption at the dopant site accompanied by individual adsorption of the other adsorbate on the pure metal surface), as determined with the DFT functionals RPBE, PBE, PBEsol and optB86b-vdW and with the foundation model MACE-MP-0. The standard deviations, σ, only take into account the DFT results. A "/" indicates an unstable or unconsidered configuration.

		Cu	Y	Zr	Nb	Mo	Tc	Ru	Rh	Pd
ΔE_{coads}(CO&CO)	RPBE	/	–9	–32	–45	–54	–55	–40	–15	3
	PBE	/	–8	–35	–48	–57	–59	–44	–20	–3
	PBEsol	/	/	–40	–53	–62	–65	–51	–28	–11
	optB86b-vdW	/	–11	–39	–51	–60	–62	–46	–23	–7
	MACE-MP-0	/	–43	–40	–33	–15	–60	–50	–32	–29
	σ	/	1	3	3	3	4	4	5	5
ΔE_{coads}(CO&H)	RPBE	/	–34	–40	–69	–88	–96	–89	–62	–31
	PBE	/	–41	–43	–71	–93	–99	–91	–64	–36
	PBEsol	/	/	–56	–75	–96	–102	–95	–69	–44
	optB86b-vdW	/	–42	–44	–69	–90	–96	–89	–62	–35
	MACE-MP-0	/	–84	–43	–29	–18	–62	–74	–60	–42
	σ	/	4	6	2	3	3	3	3	5

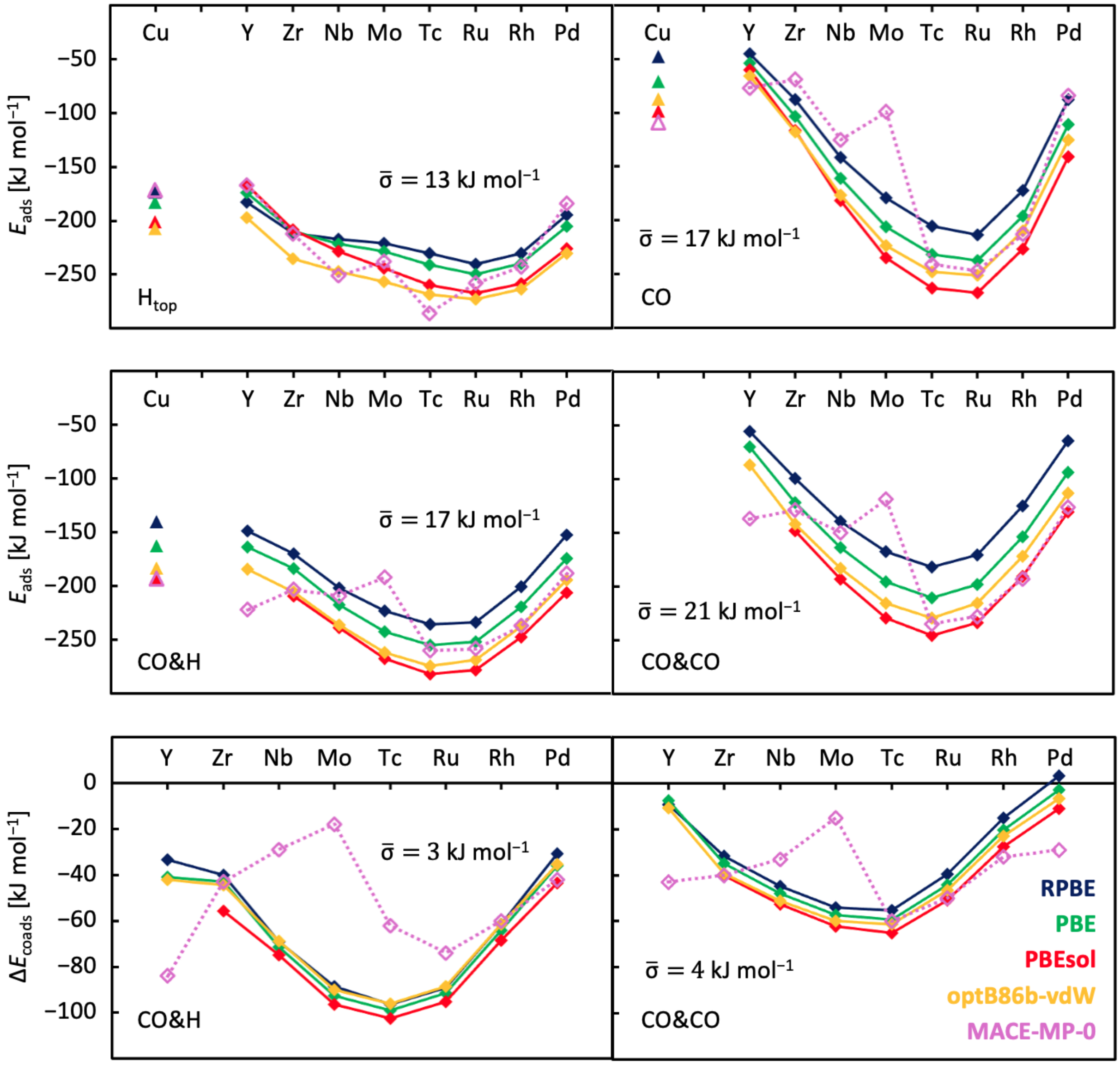

Figure S2: Adsorption energies, E_{ads}, for the individual adsorption of CO and H in atop position as well as adsorption energies and preference for coadsorption, $\Delta E_{\mathrm{coads}}$, for CO&CO and CO&H coadsorption at the same site on the Cu(111) surface, in kJ mol^{-1} per adsorbate, as determined with the DFT functionals RPBE, PBE, PBEsol and optB86b-vdW and with the foundation model MACE-MP-0. The standard deviations, σ, only take into account the DFT results. Color code: RPBE – dark blue, PBE – green, PBEsol – red, optB86b-vdW – orange, MACE-MP-0 – pink.

S5 Many-body Decomposition of the Adsorption Energy

The total energy of a system, E_{ijk}, consisting of three bodies, such as in the case of coadsorption at one site where the three bodies are the metal surface, M, and the two individual adsorbates, A and B, can be exactly expressed using the many-body decomposition, as shown in Equations (S1), (S2), and (S3).

$$E_{ijk} = \sum_{i=1}^{3} E_i^{\mathrm{1B}} + \sum_{i=1}^{3} \sum_{j>i}^{3} \Delta E_{ij}^{\mathrm{2B}} + \sum_{i=1}^{3} \sum_{j>i}^{3} \sum_{k>j}^{3} \Delta E_{ijk}^{\mathrm{3B}} \tag{S1}$$

$$\Delta E_{ij}^{\mathrm{2B}} = E_{ij}^{\mathrm{2B}} - E_i^{\mathrm{1B}} - E_j^{\mathrm{1B}} \tag{S2}$$

$$\Delta E_{ijk}^{\mathrm{3B}} = E_{ijk}^{\mathrm{3B}} - \Delta E_{ij}^{\mathrm{2B}} - \Delta E_{ik}^{\mathrm{2B}} - \Delta E_{jk}^{\mathrm{2B}} - E_i^{\mathrm{1B}} - E_j^{\mathrm{1B}} - E_k^{\mathrm{1B}} \tag{S3}$$

This decomposition is based on one-, two-, and three-body terms. The one-body terms, E_i^{1B}, represent the energies of the three individual bodies in the structure they adopt in the coadsorption motif. The two-body corrections, $\Delta E_{ij}^{\mathrm{2B}}$, are interaction energies derived from the energies of the two-body system, E_{ij}^{2B}, and the corresponding one-body energies, E_i^{1B} and E_j^{1B}. The three-body corrections, $\Delta E_{ijk}^{\mathrm{3B}}$, account for the residual interaction energy not captured by pairwise interactions. They are determined by the energy of the complete three-body system, E_{ijk}^{3B}, in conjunction with all two-body corrections and one-body energies.

Adsorption energies, E_{ads}, can be formulated using the energy of the coadsorption motif of the system comprising the three bodies A, B, and M (e.g., adsorbate A, adsorbate B, and metal surface M), $E(\mathrm{ABM})$, as well as the energies of the gas-phase equilibrium structures of the adsorbates, $E(\mathrm{A})$ and $E(\mathrm{B})$, and the equilibrium structure of the bare unloaded meta surface, $E(\mathrm{M})$, as shown in Equation (S4). By employing the many-body decomposition for the system involving the three bodies A, B, and M, $E(\mathrm{ABM})$, the adsorption energy can be represented in terms of one-body contributions (distortion energies – E_{dist}), two-body

contributions (lateral interactions – E_{lat}, adsorbate-substrate interactions – $E_{\text{ads-sub}}$), and higher-order three-body contributions (E_{3B}), see Equations (S5) to (S8).

$$E_{\text{ads}} = E(\text{ABM}) - E(\text{A}) - E(\text{B}) - E(\text{M}) = E_{\text{dist}} + E_{\text{lat}} + E_{\text{ads-sub}} + E_{\text{3B}} \tag{S4}$$

$$E_{\text{dist}} = E^{\text{1B}}_{\text{dist-A}} + E^{\text{1B}}_{\text{dist-B}} + E^{\text{1B}}_{\text{dist-M}} = E(\text{A}_{//}) - E(\text{A}) + E(\text{B}_{//}) - E(\text{B}) + E(\text{M}_{//}) - E(\text{M}) \tag{S5}$$

$$E_{\text{lat}} = \Delta E^{\text{2B}}_{\text{AB}} = E(\text{AB}_{//}) - E(\text{A}_{//}) - E(\text{B}_{//}) \tag{S6}$$

$$E_{\text{ads-sub}} = \Delta E^{\text{2B}}_{\text{AM}} + \Delta E^{\text{2B}}_{\text{BM}} = E(\text{AM}_{//}) - E(\text{A}_{//}) - E(\text{M}_{//}) + E(\text{BM}_{//}) - E(\text{B}_{//}) - E(\text{M}_{//}) \tag{S7}$$

$$E_{\text{3B}} = \Delta E^{\text{3B}}_{\text{ABM}} = E(\text{ABM}) - E(\text{AB}_{//}) - E(\text{AM}_{//}) - E(\text{BM}_{//}) + E(\text{A}_{//}) + E(\text{B}_{//}) + E(\text{M}_{//}) \tag{S8}$$

The '//' stands for 'at the structure of the coadsorption motif'. The distortion energies, E_{dist}, describe the energy change of each individual body due to the structural response upon adsorption. These calculations require the energies of the equilibrium structures of the separated bodies, $E(\text{A})$, $E(\text{B})$, and $E(\text{M})$, as well as the energies of the three separated bodies at the structures they adopt in the coadsorption motif, $E(\text{A}_{//})$, $E(\text{B}_{//})$, and $E(\text{M}_{//})$. For the calculation of lateral interactions, E_{lat}, such as those between the two bodies A and B, the energy of the two-body system at the structures of the coadsorption motif, $E(\text{AB}_{//})$, is additionally required. For the adsorbate-substrate interactions, the energies of the two systems consisting of one adsorbate and the metal surface at the structure of the coadsorption motif, $E(\text{AM}_{//})$ and $E(\text{BM}_{//})$, are needed.

Figure 4 and S3 show the many-body contributions for the coadsorption of CO&CO, CO&NO, and NO&NO at the same site on Cu(111) and Ag(111) SAAs.

The total adsorption energy for coadsorption on a given SAA is decomposed into the four contributions mentioned above, which can be opposite in direction. This analysis re-

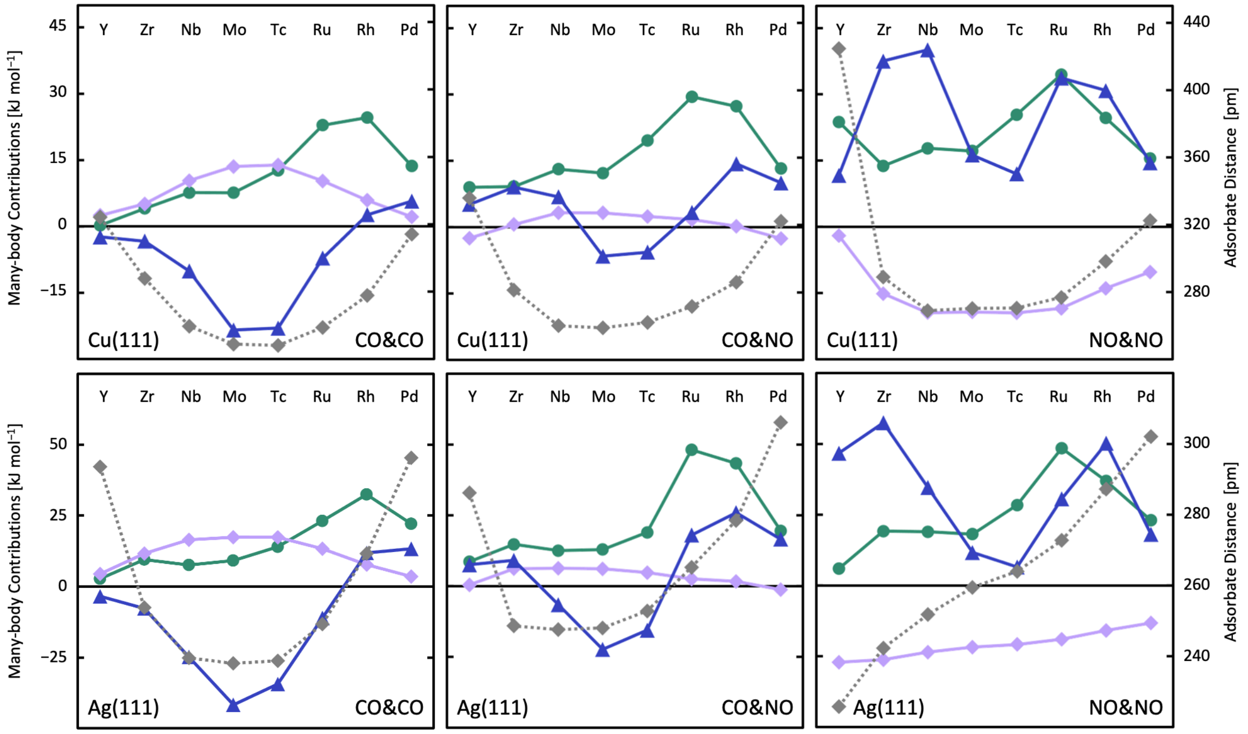

Figure S3: Many-body contributions to the adsorption energies in kJ mol^{-1} per adsorbate and distances between adsorbates in pm for the coadsorption of CO&CO, CO&NO, and NO&NO at the same site on Cu(111) and Ag(111) SAAs. Color code: green – summed one-body contributions (distortion energies), purple – two-body contributions (lateral adsorbate interaction), blue – three-body contributions, gray – adsorbate distances.

veals that most of the adsorption energy originates from the adsorbate-substrate interactions (yellow bars in Figure 4 in the main text, which explain 90% of the variation of adsorption energies (R^2 = 0.8964, Figure S4) and account for 91 to 182% (209% considering 5d TM dopants) of their magnitude. For adsorbate-substrate contributions exceeding 100%, the remaining contributions, i.e. the one-body distortion energies, lateral adsorbate-adsorbate interactions, and three-body interactions, weaken the adsorption strength. This tends to be the case for late TM SAAs, which exhibit the most pronounced distortion energies (green bars in Figure S3), e.g., for SAAs with Ru and Rh dopants. Adsorbate-substrate contributions of less than 100% indicate that the other contributions have an overall strengthening effect on adsorption. This is usually observed for small distortion energies and a combined stabilizing effect of the lateral adsorbate-adsorbate interactions (purple bars in Figure S3)

and the higher-order three-body contribution (blue bars in Figure S3), as seen, e.g., for CO coadsorption on Mo- and Tc-containing SAAs.

The distance between two adsorbates that are coadsorbed at a dopant site varies with the dopant and the host element and is a crucial factor influencing the strength of the lateral interactions. As expected, the strength of the lateral interactions increases as the distance between the adsorbates decreases, see Figure S3. In all systems except NO coadsorption on Ag-based SAAs, the adsorbates are closer to each other at central dopant elements. On the Ag-based SAAs, however, the NO-NO distance is shortest for early TMs and increases monotonically with an increasing atomic number. Thus, SAAs with an early TM dopant and an Ag(111) host surface hold promise and - again - should be further investigated as new catalysts for the formation of precursors critical for NO reduction to N_2.[S25]

Figure S4 shows the linear correlation ($R^2 = 0.8964$) between the adsorption energies for coadsorption at the same site and the sum of the adsorbate-substrate energy contributions and Figure S5 shows the qualitatively counteracting behavior of the higher order three-body and lateral two-body contributions ($R^2 = 0.5581$).

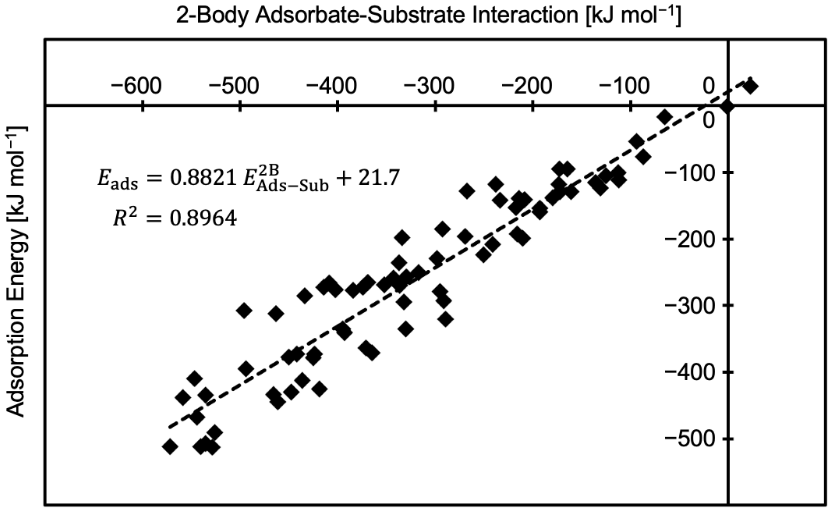

Figure S4: Correlation of adsorption energies, E_{ads}, and two-body adsorbate-substrate interaction energies, $E_{\text{ads-aub}}$, in kJ mol^{-1} obtained using a many-body decomposition for the coadsorption of CO&CO, CO&NO, and NO&NO on Cu(111) and Ag(111) SAAs, see Table S15 to S20 for the used data.

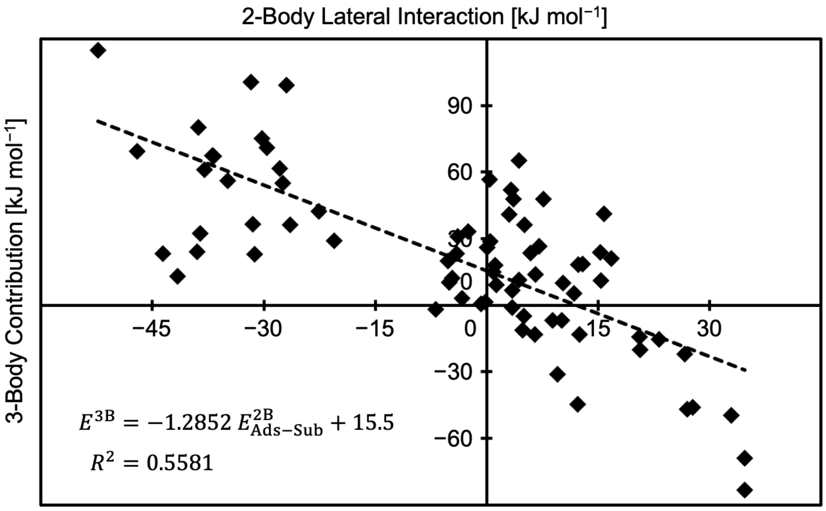

Figure S5: Correlation of three-body, E_{3B}, and lateral two-body interaction energies, E_{lat}, in kJ mol^{-1} obtained using a many-body decomposition for the coadsorption of CO&CO, CO&NO, and NO&NO on Cu(111) and Ag(111) SAAs, see Table S15 to S20 for the used data.

To investigate whether the interaction of a second adsorbate is synergistically enhanced by the first adsorption, we calculate the stepwise interaction energies, which are defined as follows:

$$E_{1.\text{int}} = E(\text{AM}_{//}) - E(\text{A}_{//}) - E(\text{M}_{//}) \tag{S9}$$

$$E_{2.\text{int}} = E(\text{ABM}) - E(\text{B}_{//}) - E(\text{AM}_{//}) \tag{S10}$$

We calculate interaction energies and not adsorption energies - the distortion energy is not considered. This eliminates any effects due to structural changes upon adsorption in the analysis. The sequential adsorption energies - including the distortion energies - can, however, be directly determined from the adsorption energies for single and co-adsorption shown in Tables S1 to S4. The interaction energy for the first adsorbate, $E_{1.\text{int}}$, is calculated using the energy of adsorbate A adsorbed at the dopant site, $E(\text{AM}_{//})$, the same adsorbate in vacuum, $E(\text{A}_{//})$, and the bare SAA surface, $E(\text{M}_{//})$, all at the unrelaxed structure of the coadsorption motif. The interaction energy for the second adsorbate, $E_{2.\text{int}}$, is calculated using the energy of both adsorbates coadsorbed at the dopant site, $E(\text{ABM})$, the second adsorbate in vacuum, $E(\text{B}_{//})$, and the first adsorbate at the dopant site, $E(\text{AM}_{//})$, all at the structure of the coadsorption motif.

While the interaction energies for adsorption of the first and second CO molecules are generally very similar, adsorption of the second CO molecule is enhanced for Nb, Mo, and Tc dopants. However, the trend reverses for the later TMs Ru, Rh, and Pd, see Tables S15 to S19 . This is consistent with the trends observed for CO coadsorption on Cu-based SAA containing 3d-TM dopants,[S26] which exhibit the same behavior for the corresponding 3d-TM dopants, i.e. an enhancement for V, Cr and Mn and a reverse trend for Fe, Co and Ni. These trends can be explained by the 10-electron rule,[S20] which proposes an optimal number of electrons that achieves maximum stabilization at dopant sites. Late TM dopants

cause a stronger adsorption of the first CO molecule, as the electrons provided by a single CO molecule bring these systems close to or already exceed the optimal electron count. The addition of a second CO molecule is less stabilizing as it may exceed the optimum value of electrons. In contrast, for early TM SAAs, the electrons provided by both CO molecules bring these electron-poor TM dopants closer to the optimal value of 10 electrons.

Table S15: Adsorption energies, E_{ads}, and contributions to the many-body decomposition, i.e, distortion energies, E_{dist}, the adsorbate-substrate, $E_{ads\text{-}sub}$, and the lateral interactions between the adsorbates, E_{lat}, as well as the higher-order three-body contributions, E_{3B}, and the interaction energies for the sequential addition of the first, $E_{1.int}$ and the second adsorbate, $E_{2.int}$ for the coadsorption of CO&CO at the same site on Cu(111)-based SAAs are given in kJ mol^{-1}. A "/" indicates an unstable or unconsidered configuration.

TM	E_{ads}	E_{dist}	E_{lat}	$E_{ads\text{-}sub}$	E_{3B}	$E_{1.int}$	$E_{2.int}$
Ni	–196	47	15	–270	11	–135	–108
Y	–112	0	5	–112	–5	–56	–56
Zr	–199	8	10	–211	–7	–105	–102
Nb	–279	15	21	–295	–20	–148	–147
Mo	–335	15	27	–330	–47	–165	–185
Tc	–364	25	28	–371	–46	–186	–204
Ru	–341	46	21	–393	–14	–197	–190
Rh	–251	49	12	–317	5	–158	–141
Pd	–129	27	4	–172	11	–86	–70
Ir	–277	76	13	–384	19	–192	–161
Pt	–139	46	6	–214	24	–107	–78

Table S16: Adsorption energies, E_{ads}, and contributions to the many-body decomposition, i.e, distortion energies, E_{dist}, the adsorbate-substrate, $E_{\text{ads-sub}}$, and the lateral interactions between the adsorbates, E_{lat}, as well as the higher-order three-body contributions, E_{3B}, and the interaction energies for the sequential addition of the first, $E_{\text{1.int}}$ and the second adsorbate, $E_{\text{2.int}}$ for the coadsorption of NO&NO at the same site on Cu(111)-based SAAs are given in kJ mol^{-1}. A "/" indicates an unstable or unconsidered configuration.

TM	E_{ads}	E_{dist}	E_{lat}	$E_{\text{ads-sub}}$	E_{3B}	$E_{\text{1.int}}$	$E_{\text{2.int}}$
Ni	–257	52	–35	–330	56	–165	–144
Y	–270	47	–4	–337	23	–162	–156
Zr	–378	28	–30	–450	75	–225	–180
Nb	–467	36	–39	–544	80	–272	–231
Mo	–507	34	–39	–535	32	–268	–273
Tc	–490	51	–39	–526	24	–264	–278
Ru	–395	69	–37	–494	67	–247	–217
Rh	–269	49	–28	–352	62	–176	–142
Pd	–153	31	–20	–193	29	–96	–88
Ir	–273	60	–30	–374	71	–187	–146
Pt	–153	45	–23	–218	42	–109	–89

Table S17: Adsorption energies, E_{ads}, and contributions to the many-body decomposition, i.e, distortion energies, E_{dist}, the adsorbate-substrate, $E_{\text{ads-sub}}$, and the lateral interactions between the adsorbates, E_{lat}, as well as the higher-order three-body contributions, E_{3B}, for the coadsorption of CO&NO at the same site on Cu(111)-based SAAs are given in kJ mol^{-1}. A "/" indicates an unstable or unconsidered configuration.

TM	E_{ads}	E_{dist}	E_{lat}	$E_{\text{ads-sub}}$	E_{3B}
Ni	–229	43	0	–298	26
Y	–193	18	–5	–216	10
Zr	–295	18	1	–332	18
Nb	–379	26	7	–425	14
Mo	–430	24	6	–447	–13
Tc	–433	39	5	–466	–11
Ru	–373	59	3	–442	7
Rh	–259	55	0	–343	29
Pd	–138	27	–5	–180	20
Ir	–276	82	3	–403	41
Pt	–141	41	–4	–209	31

Table S18: Adsorption energies, E_{ads}, and contributions to the many-body decomposition, i.e, distortion energies, E_{dist}, the adsorbate-substrate, $E_{\text{ads-sub}}$, and the lateral interactions between the adsorbates, E_{lat}, as well as the higher-order three-body contributions, E_{3B}, and the interaction energies for the sequential addition of the first, $E_{\text{1.int}}$ and the second adsorbate, $E_{\text{2.int}}$ for the coadsorption of CO&CO at the same site on Ag(111)-based SAAs are given in kJ mol^{-1}. A "/" indicates an unstable or unconsidered configuration.

TM	E_{ads}	E_{dist}	E_{lat}	$E_{\text{ads-sub}}$	E_{3B}	$E_{\text{1.int}}$	$E_{\text{2.int}}$
Ni	–185	70	17	–293	21	–146	–109
Y	–123	5	9	–131	–7	–65	–64
Zr	–224	19	23	–251	–15	–124	–119
Nb	–293	15	33	–292	–50	–146	–163
Mo	–320	18	35	–290	–83	–144	–194
Tc	–371	28	35	–365	–69	–182	–217
Ru	–373	46	7	–424	–22	–212	–207
Rh	–265	65	15	–369	24	–185	–145
Pd	–95	44	7	–173	27	–86	–53
Ir	–312	94	16	–463	41	–232	–174
Pt	–128	84	8	–267	48	–134	–78

Table S19: Adsorption energies, E_{ads}, and contributions to the many-body decomposition, i.e, distortion energies, E_{dist}, the adsorbate-substrate, $E_{\text{ads-sub}}$, and the lateral interactions between the adsorbates, E_{lat}, as well as the higher-order three-body contributions, $E_{3\text{B}}$, and the interaction energies for the sequential addition of the first, $E_{1.\text{int}}$ and the second adsorbate, $E_{2.\text{int}}$ for the coadsorption of NO&NO at the same site on Ag(111)-based SAAs are given in kJ mol^{-1}. A "/" indicates an unstable or unconsidered configuration.

TM	E_{ads}	E_{dist}	E_{lat}	$E_{\text{ads-sub}}$	$E_{3\text{B}}$	$E_{1.\text{int}}$	$E_{2.\text{int}}$
Ni	–236	71	–37	–337	67	–169	–139
Y	–285	12	–54	–337	93	–165	–132
Zr	–434	38	–52	–536	115	–266	–206
Nb	–512	38	–47	–572	69	–286	–264
Mo	–512	36	–44	–528	23	–264	–284
Tc	–512	57	–42	–540	13	–270	–299
Ru	–439	97	–38	–559	61	–280	–256
Rh	–266	74	–32	–409	101	–204	–136
Pd	–118	46	–26	–174	36	–87	–77
Ir	–285	76	–27	–434	99	–220	–142
Pt	–141	65	–27	–234	55	–117	–89

Table S20: Adsorption energies, E_{ads}, and contributions to the many-body decomposition, i.e, distortion energies, E_{dist}, the adsorbate-substrate, $E_{\text{ads-sub}}$, and the lateral interactions between the adsorbates, E_{lat}, as well as the higher-order three-body contributions, E_{3B}, for the coadsorption of CO&NO at the same site on Ag(111)-based SAAs are given in kJ mol^{-1}. A "/" indicates an unstable or unconsidered configuration.

TM	E_{ads}	E_{dist}	E_{lat}	$E_{\text{ads-sub}}$	E_{3B}
Ni	–198	85	4	–334	48
Y	–208	17	1	–241	15
Zr	–335	30	12	–395	18
Nb	–412	25	13	–437	–13
Mo	–425	26	12	–419	–45
Tc	–445	38	10	–461	–31
Ru	–409	96	5	–547	36
Rh	–272	87	3	–414	52
Pd	–95	39	–2	–165	33
Ir	–307	119	4	–496	65
Pt	–118	63	0	–238	57

S6 Vibrational Frequencies

We calculate the harmonic frequencies using a partial Hessian that permits the movement of the adsorbates and the dopant atom as described in Section S1, see Table S21 for the results. To empirically correct the approximations made with the harmonic approximation and a DFT-based PES, we also calculate scaled frequencies. (Table S22) The scaling takes into account the anharmonicity and the deviation of the curvature of the DFT PES from the exact PES, assuming that these are systematic errors for similar vibrations, e.g. for stretching vibrations of a particular adsorbate. The scaling factor F for a given type of vibration is defined as the ratio between the experimental frequency and the corresponding calculated harmonic frequency:

$$F = \frac{\nu_{\text{exp}}}{\nu_{\text{calc}}} \tag{S11}$$

We use a scaling factor of 1.0205 ($\nu_{\text{exp}} = 2143$,[S27] $\nu_{\text{calc}} = 2100$) for the frequency of the CO stretching vibrations, a factor of 0.9899 ($\nu_{\text{exp}} = 1876$,[S28] $\nu_{\text{calc}} = 1895$) for the NO stretching vibrations and a factor of 1.0315 ($\nu_{\text{exp}} = 1040$,[S16,S29] $\nu_{\text{calc}} = 1008$) for the stretching vibration of an H atom adsorbed on a hollow fcc site on Cu(111). Although these scaling factors correct for systematic errors, they cannot eliminate deviations in the frequencies that are due to an inaccurate description of the interactions between the adsorbates and the metal surfaces.

The scaling approach works well, as demonstrated for CO and NO adsorption on pure metal and SAA surfaces. The calculated and experimental frequencies for CO adsorbed in atop position on Cu(111) ($\nu_{\text{CO, calc}} = 2034$ cm^{-1}, $\nu_{\text{CO, exp}} = 2078$ cm^{-1}),[S30] NO adsorbed on a Cu(111) hollow fcc site ($\nu_{\text{NO, calc}} = 1459$ cm^{-1}, $\nu_{\text{NO, exp}} = 1525$ cm^{-1}),[S31] and CO adsorbed on top of the dopant in PdAg(111) ($\nu_{\text{CO, calc}} = 2021$ cm^{-1}, $\nu_{\text{CO, exp}} = 2047$ cm^{-1})[S32] are in good agreement, exhibiting deviations of 26 to 66 cm^{-1} which are typical for simulation in these systems.[S16,S17,S32–S34] For trends across the periodic table and for shifts in vibrational

frequencies from the atop adsorption motif to coadsorption at the same site, we expect even smaller deviations due to error compensation. This is evidenced by the excellent agreement of the experimental and calculated shifts of -52[S33] and -57 cm^{-1} for the CO stretching vibration frequency on RhCu(100) and RhCu(111), respectively.

Only the symmetric but not the antisymmetric combination of stretching vibrations can be observed with IR spectroscopy according to the selection rules on metallic surfaces.[S33]

Table S21: Harmonic frequencies of: (1) the stretching vibrations of individually adsorbed CO and NO in the atop position on the dopant as well as H and, if more stable, NO adsorbed at a hollow fcc site next to the dopant; (2) the symmetric (sym) and asymmetric (asym) combinations of the CO or NO stretching vibrations of the CO&CO and NO&NO coadsorption motifs; and (3) the stretching vibrations of H, CO, and NO coadsorbed at the same site in the CO&H and NO&H coadsorption motifs on the Cu(111) surface. All frequencies are reported in cm^{-1}. A "/" indicates an unstable or unconsidered configuration.

TM	H_{fcc}	CO_{atop}	NO_{atop}	NO_{fcc}	CO&CO	NO&NO	CO&H	NO&H
					sym | asym	sym | asym	ν(CO) | ν(HM)	ν(NO) | ν(HM)
Cu	1008	1993	1717	1474	/	/	1802 | 1033	1478 | 1048
Y	1013	2024	1660	/	2014 | 1956	1485 | 1365	1863 | 1020	1502 | 1026
Zr	1060	1987	1667	1560	1944 | 1885	1625 | 1552	1962 | 1074	1565 | 1015
Nb	1170	1934	1688	/	1907 | 1849	1690 | 1619	1918 | 1237	1691 | 1172
Mo	1252	1909	1733	/	1895 | 1840	1696 | 1635	1901 | 1401	1742 | 1301
Tc	1326	1903	1755	/	1895 | 1842	1707 | 1648	1900 | 1505	1766 | 1371
Ru	1409	1926	1798	/	1905 | 1855	1697 | 1638	1928 | 1519	1801 | 1340
Rh	1325	1965	1812	/	1909 | 1862	1641 | 1577	1955 | 1348	1644 | 1407
Pd	1083	2002	1779	1506	1903 | 1858	1643 | 1571	1838 | 1113	1524 | 1114
Ag	1043	2035	1737	1514	/	/	1985 | 1050	1471 | 1014

Table S22: Scaled frequencies of: (1) the stretching vibrations of individually adsorbed CO and NO in the atop position on the dopant as well as H and, if more stable, NO adsorbed at a hollow fcc site next to the dopant; (2) the symmetric (sym) and asymmetric (asym) combinations of the CO or NO stretching vibrations of the CO&CO and NO&NO coadsorption motifs; and (3) the stretching vibrations of H, CO, and NO coadsorbed at the same site in the CO&H and NO&H coadsorption motifs on the Cu(111) surface. The scaling factors for the frequencies of intramolecular stretching vibrations of CO and NO are 1.0205 and 0.9899, respectively, and 1.0315 for the stretching vibration of an adsorbed H atom. All frequencies are reported in cm^{-1}. A "/" indicates an unstable or unconsidered configuration.

TM	H_{fcc}	CO_{atop}	NO_{atop}	NO_{fcc}	CO&CO	NO&NO	CO&H	NO&H
					sym | asym	sym | asym	ν(CO) | ν(HM)	ν(NO) | ν(HM)
Cu	1040	2034	1699	1459	/	/	1839 | 1065	1463 | 1081
Y	1045	2066	1643	/	2055 | 1996	1470 | 1351	1901 | 1052	1487 | 1059
Zr	1094	2027	1650	1545	1983 | 1924	1609 | 1536	2003 | 1108	1550 | 1047
Nb	1207	1974	1671	/	1946 | 1887	1673 | 1603	1957 | 1276	1674 | 1209
Mo	1292	1948	1716	/	1934 | 1878	1678 | 1618	1940 | 1445	1725 | 1341
Tc	1368	1942	1737	/	1934 | 1879	1689 | 1632	1939 | 1552	1748 | 1414
Ru	1453	1965	1779	/	1944 | 1893	1680 | 1621	1968 | 1567	1783 | 1382
Rh	1367	2005	1794	/	1948 | 1900	1624 | 1561	1995 | 1390	1627 | 1451
Pd	1117	2043	1761	1491	1942 | 1896	1626 | 1555	1875 | 1148	1509 | 1149
Ag	1076	2077	1720	1498	/	/	2026 | 1084	1456 | 1046

Table S23: Shifts in the scaled frequencies caused by the change in adsorption motif from individual adsorption at a dopant to coadsorption at the same site for the symmetric (sym) and asymmetric (asym) combinations of the CO or NO stretching vibrations of the CO&CO and NO&NO coadsorption motifs and the stretching vibrations of H, CO and NO coadsorbed at the same site in the CO&H and NO&H coadsorption motifs on the Cu(111) surface. The scaling factors for the frequencies of intramolecular stretching vibrations of CO and NO are 1.0205 and 0.9899, respectively, and 1.0315 for the stretching vibration of an adsorbed H atom. All frequencies are reported in cm^{-1}. A "/" indicates an unstable or unconsidered configuration.

TM	CO&CO	NO&NO	CO&H	NO&H
	sym | asym	sym | asym	ν(CO) | ν(HM)	ν(NO) | ν(HM)
Cu	/	/	–195 | 25	–236 | 41
Y	–11 | –70	–173 | –292	–165 | 8	–157 | 14
Zr	–44 | –104	–41 | –114	–25 | 14	–100 | –46
Nb	–28 | –87	2 | –69	–16 | 69	3 | 2
Mo	–14 | –70	–37 | –97	–8 | 153	9 | 50
Tc	–8 | –62	–48 | –105	–3 | 184	11 | 46
Ru	–21 | –73	–99 | –158	2 | 114	4 | –71
Rh	–57 | –105	–170 | –233	–10 | 23	–167 | 84
Pd	–101 | –147	–135 | –205	–168 | 31	–252 | 32
Ag	/	/	–51 | 8	–264 | –30

S7 Kinetic Monte Carlo Simulations

The important energetics for the two model systems used in the kinetic Monte Carlo (KMC) simulations are shown in Figure 6 of the main text. A comprehensive list of relative energies between states is provided in Table S24. Barriers for all processes considered in the KMC simulations are provided in Table S25. The reaction profile is designed such that coadsorption on a pure host metal surface, A&A, constitutes a distinct, destabilized state that mimics repulsive lateral interactions. In this model, we assume this is a distinct state and hence a local minimum on the potential energy surface. While this state may not constitute a local minimum for some reactant-surface combinations, it will be for others and, in any case, serves as a valuable, albeit simplified, model for understanding factors influencing catalyst activity. In contrast, at dopant sites, this state is stabilized, reflecting the preferential coadsorption observed on SAAs. Once the reaction-ready state is reached, the barrier to forming B (highlighted in red in Figure 6 of the main text) is identical for both the pure metal surface and the SAA.

The simulated surfaces consist of (50×50) rectangular unit cells, resulting in 2500 adsorption sites per periodic simulation cell. For the SAA model, 25 dopant sites randomly replace host sites, mimicking a dopant concentration of 1%. Each site is connected to four neighboring sites. The initial coverage is set to 15%, corresponding to 375 randomly placed species A. Example input files for the models are provided as additional Supporting Information.

Table S26 summarizes the times, $t_{20\%}$, required to convert 20% (76 A) of the initially adsorbed species A (coverage 15%, 375 A) to the desorbed product B_{gas} (38 B) on pure metal and single-atom alloy (SAA) surface models at different temperatures, as obtained from eight independent KMC simulations for each temperature using randomized seeds and randomized initial coverage of species A on the surfaces.

The plot shown in Figure 6 of the main text is similar to an Arrhenius plot but does not contain the same information. While an Arrhenius plot shows how $\ln(k)$, with k being the rate constant, changes as a function of $1/T$, in our Arrhenius-like plot we use the inverse

of the time $t_{20\%}$, which is the time required to convert 20% of the initially adsorbed species A to the desorbed product B_{gas}. This inverse time can be regarded as a proxy for the rate constant, which is, however, actually a reaction rate for the initial transformation of the reactants. We chose this inverse time over the rate constant because it is a direct result of the KMC simulation, and we do not need to incorporate further knowledge about the reaction order or reactant concentration and coverage. Further, since this is only a model reaction used to assess a qualitative effect, this analysis is sufficient, considering that the ratio of the slopes of the two linearized equations is equal to the ratio of slopes of the two true Arrhenius equations under otherwise equal conditions.

While the barriers for the elementary reaction step from the coadsorption state A&A $\rightarrow$ $TS^{\ddagger}$ $\rightarrow$ B remain the same, the overall energy profile for the reaction in both models changes significantly due to the (de)stabilization of the coadsorbed state. Consequently, the time required to transform 20% of the reactants, $t_{20\%}$, is reduced by 2 to 3 orders of magnitude at a given temperature in the SAA model system. This substantial acceleration is evident as a pronounced downshift in the Arrhenius-like plot for the dimerization reaction on host metal sites (gray line and symbols in Figure 6 of the main text) compared to what is observed in the SAA model (orange line and symbols in Figure 6 of the main text).

Furthermore, the Arrhenius-like plot for the dimerization reaction on host metal sites is significantly steeper compared to that at dopant sites, indicating a higher effective activation energy for the former. According to the Arrhenius equation, the ratio of the two slopes (1.77) equals the ratio of the activation energies. This assumption holds since the systems are in the low coverage regime (initial coverage is 15%) and saturation effects should not play a significant role. The ratio of the two slopes is similar to the ratio of a combined barrier comprising the energy required to form the reaction-ready coadsorbed state $A\&A_{host}$ on a host metal site (+0.4 eV) and the barrier of the elementary step that forms B (+0.6 eV) for the pure metal catalyst divided by the barrier for the elementary reaction step on the SAA catalyst: $1.67 = (0.4 \text{ eV} + 0.6 \text{ eV}) / 0.6 \text{ eV}$.

Table S24: Relative energies between the states considered in the kinetic Monte Carlo (KMC) simulations in eV. Species A in the gas phase, A_{gas}, is defined as the reference with an energy of 0.0 eV. The other considered states are: B in the gas phase, B_{gas}; A adsorbed at a host, A_{host}, or dopant, A_{dop}, site; B adsorbed at a host, B_{host}, or dopant, B_{dop}, site; and 2 A coadsorbed at a host, $A\&A_{host}$, or dopant, $A\&A_{dop}$, site. Numbers in parentheses are normalized per species A that is needed for its formation.

	A_{gas}	B_{gas}	A_{host}	A_{dop}	$A\&A_{host}$	$A\&A_{dop}$	B_{host}	B_{dop}
pure host metal	0.0	–1.3	–0.6	/	–0.8	/	–1.6	/
	/	(–0.65)	/	/	(–0.4)	/	(–0.8)	/
single-atom alloy	0.0	–1.3	–0.6	–1.0	–0.8	–2.0	–1.6	–2.1
	/	(–0.65)	/	/	(–0.4)	(–1.0)	(–0.8)	(–1.05)

Table S25: Processes considered in the kinetic Monte Carlo (KMC) simulations, including their initial and final states, as well as the barriers in eV. Preexponential factors are set to 10^{13}. Sites i and j can be either host metal or dopant sites, and are neighboring and connected.

	initial state	final state	barrier
diffusion	$A_{host\text{-}i}$	$A_{host\text{-}j}$	0.15
	$A_{dopant\text{-}i}$	$A_{host\text{-}j}$	0.15
reactions	$A_{host\text{-}i}$, $A_{host\text{-}j}$	$A\&A_{host\text{-}i}$	0.4
	$A_{dop\text{-}i}$, $A_{host\text{-}j}$	$A\&A_{dop\text{-}i}$	0.05
	$A\&A_{host\text{-}i}$	$B_{host\text{-}i}$	0.6
	$A\&A_{dop\text{-}i}$	$B_{dop\text{-}i}$	0.6
desorption	$B_{host\text{-}i}$	B_{gas}	0.3
	$B_{dop\text{-}i}$	B_{gas}	0.8

Table S26: Time, $t_{20\%}$, required to convert 20% of the initial species A to the desorbed product B_{gas} on pure metal and single-atom alloy (SAA) surface models at different temperatures, as obtained from 8 independent and randomized kinetic Monte Carlo (KMC) simulations. Times are given in seconds. Additional settings applied: snapshots 1.0E–8, process_statistics 1.0E–9, and species_numbers 1.0E–9.

T [K]	surface	KMC-1	KMC-2	KMC-3	KMC-4	KMC-5	KMC-6	KMC-7	KMC-8
350	pure metal	/	/	/	/	/	/	/	/
	SAA	2.31E–5	2.04E–5	2.04E–5	2.10E–5	2.21E–5	2.41E–5	1.89E–5	2.35E–5
400	pure metal	/	/	/	/	/	/	/	/
	SAA	2.22E–6	2.01E–6	2.33E–6	1.81E–6	1.93E–6	2.04E–6	1.57E–6	2.03E–6
450	pure metal	/	/	/	/	/	/	/	/
	SAA	3.31E–7	2.56E–7	2.96E–7	3.68E–7	2.59E–7	2.97E–7	2.90E–7	3.74E–7
500	pure metal	/	/	/	/	/	/	/	/
	SAA	7.0E–8	6.9E–8	7.1E–8	7.9E–8	6.7E–8	6.1E–8	6.8E–8	6.5E–8
550	pure metal	/	/	/	/	/	/	/	/
	SAA	1.8E–8	2.1E–8	2.2E–8	2.4E–8	1.7E–8	2.0E–8	2.2E–8	1.9E–8
600	pure metal	/	/	/	/	/	/	/	/
	SAA[a]	6.39E–9	7.62E–9	6.99E–9	5.91E–9	7.39E–9	6.37E–9	7.08E–9	9.45E–9
650	pure metal	4.99E–6	6.51E–6	3.88E–6	3.70E–6	4.68E–6	5.75E–6	5.04E–6	4.48E–6
	SAA[a]	3.07E–9	3.09E–9	3.19E–9	2.37E–9	3.09E–9	3.55E–9	3.68E–9	3.10E–9
700	pure metal	9.94E–7	1.04E–6	1.13E–6	9.74E–7	1.16E–6	1.19E–6	8.30E–7	1.38E–6
	SAA[a]	1.55E–9	1.45E–9	1.19E–9	1.27E–9	1.52E–9	1.08E–9	1.56E–9	1.58E–9
750	pure metal	3.27E–7	3.64E–7	3.75E–7	4.28E–7	3.00E–7	3.39E–7	4.44E–7	4.03E–7
	SAA[a]	7.2E–10	6.7E–10	5.8E–10	7.8E–10	8.2E–10	6.6E–10	7.2E–10	7.0E–10
800	pure metal	1.64E–7	1.53E–7	1.36E–7	1.89E–7	1.43E–7	1.07E–7	1.47E–7	2.10E–7
	SAA[a]	3.9E–10	4.0E–10	3.2E–10	4.2E–10	3.3E–10	4.9E–10	4.2E–10	4.1E–10
850	pure metal	5.4E–8	6.5E–8	7.4E–8	6.0E–8	5.9E–8	4.2E–8	6.9E–8	5.5E–8
	SAA[a]	2.9E–10	2.5E–10	2.7E–10	2.5E–10	2.1E–10	2.4E–10	2.5E–10	2.4E–10
900	pure metal	2.1E–8	2.8E–8	3.0E–8	2.9E–8	2.4E–8	3.3E–8	3.0E–8	2.4E–8
	SAA[a]	1.7E–10	2.1E–10	1.5E–10	1.6E–10	2.2E–10	1.9E–10	1.6E–10	2.0E–10

[a] snapshots 1.0E–10, process_statistics 1.0E–11, species_numbers 1.0E–11

References

(S1) Hammer, B.; Hansen, L. B.; Nørskov, J. K. Improved adsorption energetics within density-functional theory using revised Perdew-Burke-Ernzerhof functionals. *Phys. Rev. B.* **1999**, *59*, 7413.

(S2) Kresse, G.; Hafner, J. Ab initio molecular dynamics for liquid metals. *Phys. Rev. B* **1993**, *47*, 558.

(S3) Kresse, G.; Furthmüller, J. Efficiency of ab-initio total energy calculations for metals and semiconductors using a plane-wave basis set. *Comput. Mater. Sci.* **1996**, *6*, 15.

(S4) Kresse, G.; Furthmüller, J. Efficient iterative schemes for ab initio total-energy calculations using a plane-wave basis set. *Phys. Rev. B* **1996**, *54*, 11169.

(S5) Kresse, G.; Joubert, D. From ultrasoft pseudopotentials to the projector augmented-wave method. *Phys. Rev. B* **1999**, *59*, 1758.

(S6) Perdew, J. P.; Burke, K.; Ernzerhof, M. Generalized Gradient Approximation Made Simple. *Phys. Rev. Lett.* **1996**, *77*, 3865.

(S7) Perdew, J. P.; Ruzsinszky, A.; Csonka, G. I.; Vydrov, O. A.; Scuseria, G. E.; Constantin, L. A.; Zhou, X.; Burke, K. Restoring the Density-Gradient Expansion for Exchange in Solids and Surfaces. *Phys. Rev. Lett.* **2008**, *100*, 136406.

(S8) Perdew, J. P.; Ruzsinszky, A.; Csonka, G. I.; Vydrov, O. A.; Scuseria, G. E.; Constantin, L. A.; Zhou, X.; Burke, K. Erratum: Restoring the Density-Gradient Expansion for Exchange in Solids and Surfaces [Phys. Rev. Lett. 100, 136406 (2008)]. *Phys. Rev. Lett.* **2009**, *102*, 039902.

(S9) Klimeš, J.; Bowler, D. R.; Michaelides, A. Van der Waals density functionals applied to solids. *Phys. Rev. B* **2011**, *83*, 195131.

(S10) Alchagirov, A. B.; Perdew, J. P.; Boettger, J. C.; Albers, R.; Fiolhais, C. Reply to "Comment on 'Energy and pressure versus volume: Equations of state motivated by the stabilized jellium model'". *Phys. Rev. B* **2003**, *67*, 026103.

(S11) Stamatakis, M.; Vlachos, D. G. A graph-theoretical kinetic Monte Carlo framework for on-lattice chemical kinetics. *J. Chem. Phys.* **2011**, *134*.

(S12) Savva, G. D.; Stamatakis, M. Comparison of queueing data-structures for kinetic Monte Carlo simulations of heterogeneous catalysts. *J. Phys. Chem. A* **2020**, *124*, 7843.

(S13) Ravipati, S.; d'Avezac, M.; Nielsen, J.; Hetherington, J.; Stamatakis, M. A caching scheme to accelerate kinetic Monte Carlo simulations of catalytic reactions. *J. Phys. Chem. A* **2020**, *124*, 7140.

(S14) Pineda, M.; Stamatakis, M. Kinetic Monte Carlo simulations for heterogeneous catalysis: Fundamentals, current status, and challenges. *J. Chem. Phys.* **2022**, *156*.

(S15) Zheng, H.; Li, H.; Luo, L.; Zhao, Z.; Henkelman, G. Factors that influence hydrogen binding at metal-atop sites. *J. Chem. Phys.* **2021**, *155*, 024703.

(S16) Xu, L.; Lin, J.; Bai, Y.; Mavrikakis, M. Atomic and molecular adsorption on Cu (111). *Top. Catal.* **2018**, *61*, 736.

(S17) Chen, B. W.; Kirvassilis, D.; Bai, Y.; Mavrikakis, M. Atomic and molecular adsorption on Ag (111). *J. Phys. Chem. C* **2018**, *123*, 7551.

(S18) Abild-Pedersen, F.; Andersson, M. P. CO adsorption energies on metals with correction for high coordination adsorption sites–A density functional study. *Surf. Sci.* **2007**, *601*, 1747.

(S19) Feibelman, P. J.; Hammer, B.; Nørskov, J. K.; Wagner, F.; Scheffler, M.; Stumpf, R.; Watwe, R.; Dumesic, J. The co/pt (111) puzzle. *J. Phys. Chem. B* **2001**, *105*, 4018.

(S20) Schumann, J.; Stamatakis, M.; Michaelides, A.; Réocreux, R. Ten-electron count rule for the binding of adsorbates on single-atom alloy catalysts. *Nat. Chem.* **2024**, *16*, 749.

(S21) Schimka, L.; Harl, J.; Stroppa, A.; Grüneis, A.; Marsman, M.; Mittendorfer, F.; Kresse, G. Accurate surface and adsorption energies from many-body perturbation theory. *Nat. Mater.* **2010**, *9*, 741.

(S22) Batatia, I.; Benner, P.; Chiang, Y.; Elena, A. M.; Kovács, D. P.; Riebesell, J.; Advincula, X. R.; Asta, M.; Baldwin, W. J.; Bernstein, N.; others A foundation model for atomistic materials chemistry. *arXiv preprint arXiv:2401.00096* **2023**,

(S23) Batatia, I.; Kovacs, D. P.; Simm, G.; Ortner, C.; Csányi, G. MACE: Higher order equivariant message passing neural networks for fast and accurate force fields. *Adv. Neural. Inf. Process. Syst.* **2022**, *35*, 11423.

(S24) Jain, A.; Ong, S. P.; Hautier, G.; Chen, W.; Richards, W. D.; Dacek, S.; Cholia, S.; Gunter, D.; Skinner, D.; Ceder, G.; others Commentary: The Materials Project: A materials genome approach to accelerating materials innovation. *APL Mater.* **2013**, *1*.

(S25) Xing, F.; Jeon, J.; Toyao, T.; Shimizu, K.-i.; Furukawa, S. A Cu–Pd single-atom alloy catalyst for highly efficient NO reduction. *Chem. Sci.* **2019**, *10*, 8292.

(S26) Christiansen, M. A.; Peña-Torres, A.; Jonsson, E. O.; Jónsson, H. Single-Atom Substituents in Copper Surfaces May Adsorb Multiple CO Molecules. *J. Phys. Chem. Lett.* **2024**, *15*, 5654.

(S27) London, J. W.; Bell, A. T. Infrared spectra of carbon monoxide, carbon dioxide, nitric oxide, nitrogen dioxide, nitrous oxide, and nitrogen adsorbed on copper oxide. *J. Catal.* **1973**, *31*, 32.

(S28) Drouet, C.; Alphonse, P.; Rousset, A. IR spectroscopic study of NO and CO adsorptions on nonstoichiometric nickel–copper manganites. *Phys. Chem. Chem. Phys.* **2001**, *3*, 3826.

(S29) Lee, G.; Plummer, E. High-resolution electron energy loss spectroscopy study on chemisorption of hydrogen on Cu (111). *Surf. Sci.* **2002**, *498*, 229.

(S30) Eve, J.; McCash, E. Low-temperature adsorption of CO on Cu (1 1 1) studied by RAIRS. *Chem. Phys. Lett.* **2002**, *360*, 202.

(S31) Dumas, P.; Suhren, M.; Chabal, Y.; Hirschmugl, C.; Williams, G. Adsorption and reactivity of NO on Cu (111): a synchrotron infrared reflection absorption spectroscopic study. *Surf. Sci.* **1997**, *371*, 200.

(S32) Muir, M.; Trenary, M. Adsorption of CO to characterize the structure of a Pd/Ag (111) single-atom alloy surface. *J. Phys. Chem. C* **2020**, *124*, 14722.

(S33) Wang, Y.; Schumann, J.; Happel, E. E.; Çınar, V.; Sykes, E. C. H.; Stamatakis, M.; Michaelides, A.; Hannagan, R. T. Observation and Characterization of Dicarbonyls on a RhCu Single-Atom Alloy. *J. Phys. Chem. Lett.* **2022**, *13*, 6316.

(S34) Therrien, A. J.; Hensley, A. J.; Marcinkowski, M. D.; Zhang, R.; Lucci, F. R.; Coughlin, B.; Schilling, A. C.; McEwen, J.-S.; Sykes, E. C. H. An atomic-scale view of single-site Pt catalysis for low-temperature CO oxidation. *Nat. Catal.* **2018**, *1*, 192.
